# A systematic review of advances in preparation, structures, bioactivities, structural-property relationships, and applications of *Polyporus umbellatus* polysaccharides

**DOI:** 10.1016/j.fochx.2025.102161

**Published:** 2025-01-06

**Authors:** Wei Gao, Yongbin Xu, Weihao Chen, Jianjun Wu, Yu He

**Affiliations:** School of Pharmaceutical Sciences, Zhejiang Chinese Medical University, Hangzhou 311402, China

**Keywords:** *Polyporus umbellatus*, Polysaccharides, Preparation, Structural characterization, Structure-activity relationships

## Abstract

*Polyporus umbellatus* (Pers.) Fries is an edible fungus species belonging to the Polygonaceae family. Polysaccharides, the predominant bioactive compounds in *P. umbellatus*, have been widely used due to its abundant nutritional and medicinal benefits. Since the first unrefined *P. umbellatus* polysaccharides (PUPs) was obtained in 1973, they have been studied for half a century, and are currently gaining increasing attention. These research findings are however quite fragmented. In this review, current relevant research data regarding techniques for the preparation (extraction, fractionation, and purification) and structural characterization (molecular weight, monosaccharide composition, glycosidic bond types, and structural features) of PUPs covering a period of over 50 years are reviewed. Furthermore, this review comprehensively examines the functional properties, structure-activity relationships, and current applications of PUPs. Future research should prioritize standardized preparation process, reliable quality control and specific mechanisms to further advance the utilization and development of PUPs and their related products.

## Highlights

• *Polyporus umbellatus* polysaccharides (PUPs) are currently gaining increasing attention.
• Techniques for the preparation and structural characterization of PUPs are reviewed.
• The functional properties and application status of PUPs are also reviewed.
• The relationship of structural characteristics and functional properties of PUPs are discussed.
• PUPs have a broad application prospect in functional food, medicine, and cosmetic fields.

## 1 Introduction

**Fig. 1:**
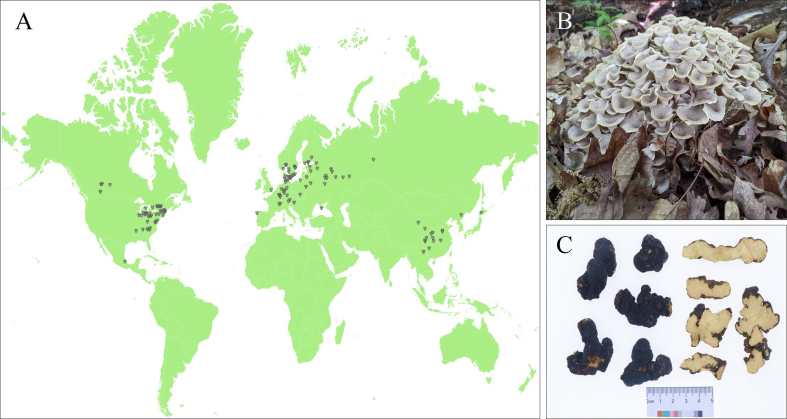
The major distribution of *P. umbellatus* in the world (A), the fruiting body of *P. umbellatus* (B), the sclerotia of *P. umbellatus* (C).

**Fig. 2:**
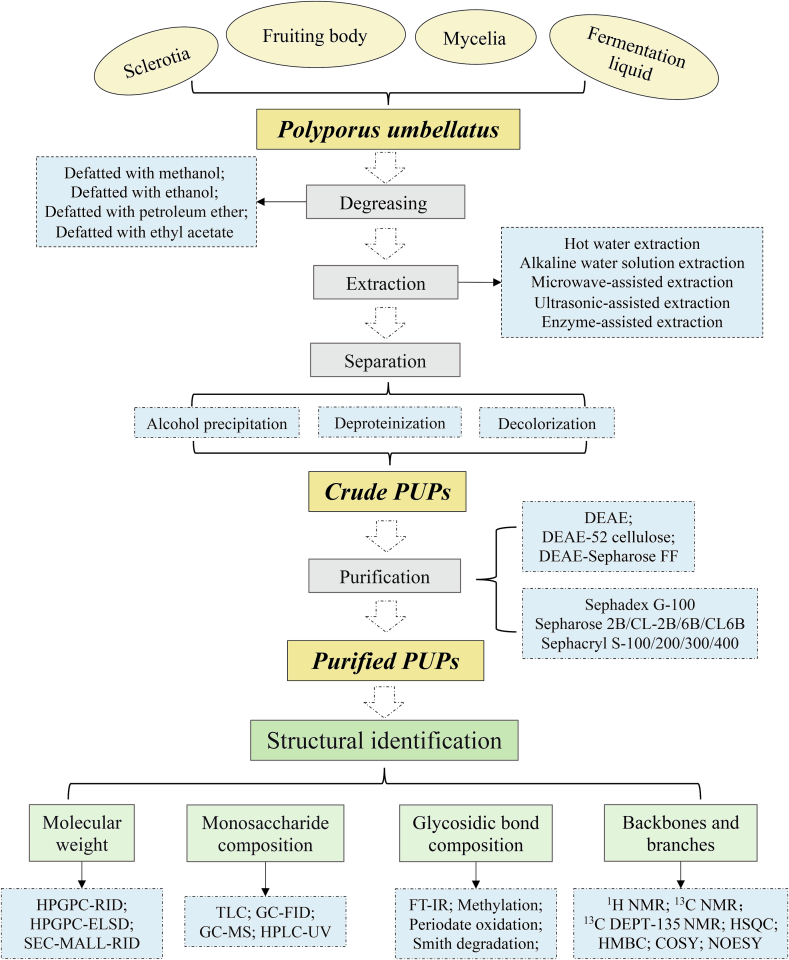
Procedures for extraction, purification, and structural identification of PUPs.

**Fig. 3:**
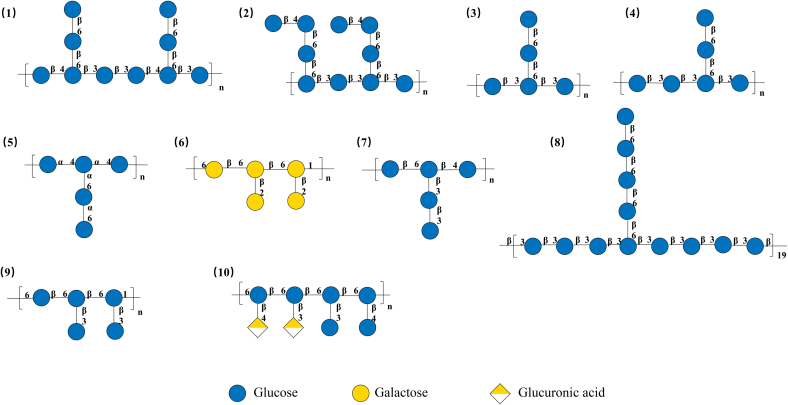
Proposed structure of the repeating unit for well characterized *P. umbellatus* polysaccharides. GU-1 (1) (2), ZP (3) (4), HPP (5), PPUS and PUP60W-1 (6), ZPS (7), PUP-W-1 (8), PUP80S1 (9), PUP60S2 (10).

*Polyporus umbellatus* (Pers.) Fries, is a perennial lignicolous fungus belonging to the Polyporaceae family (Basidiomycotina). It has a wide distribution in temperate oak forests and is associated with broad-leaf, coniferous, and mixed coniferous forests mostly found in altitudes ranging between 800 and 1500 m (Fig. 1A) (Dai, 2012; Guo et al., 2018). *P. umbellatus* has a long history as a source of food and medicine. It has two parts, the underground portion of the sclerotia and the above-ground portion which is the fruiting body (Fig. 1B) (iNaturalist, 2023; Wang, Guo, Fan, & Xue, 2004). The sclerotia has been documented in the “Shen Nong's Canon of Materia Medica”, which dates back to approximately 2000 years ago, to have been used for medicinal purposes (Fig. 1C). It was also officially included in the 2020 edition of the Chinese Pharmacopoeia, as a medication with therapeutic applications in treating dampness, diuresis and reducing swelling. In 2018, the safety and health of *P. umbellatus* were evaluated and subsequently certified as an edible organic product by the China Accreditation Administration (CNCA). This recognition further solidified its status as an edible product within the framework of the Organic Product Certification Program. (See Fig. 2, Fig. 3.)

*P. umbellatus* has evolved intricate biochemical pathways capable of producing a wide array of metabolites including, polysaccharides (Bi, Miao, Han, Xu, & Wang, 2013; Dai et al., 2012; Li et al., 2019; Liu, Li, Song, Zhang, & Wang, 2019; Sun & Zhou, 2014; Zhao, 2013), steroids (Sun & Yasukawa, 2008; Wang, Wu, Zhang, Hu, & Miao, 2021; Zhao, 2013; Zhou, Lin, & Guo, 2007), anthraquinones (Zhou & Guo, 2009), triterpenoids (Zhao et al., 2011; Zhou & Guo, 2009), amino acids (Yang, 2000), inorganic elements (Ge, Wang, & Zhang, 2016; Yang, 2000), vitamins (Yang, 2000), and trace minerals (Yang, 2000). *In vitro* and *in vivo* pharmacological experiments and clinical practice have proved, each exhibiting an array of biological activities. Among these, the *P. umbellatus* polysaccharides (PUPs) are considered the primary components responsible for the bioactivities observed. PUPs are currently being utilized in the creation of biopharmaceuticals, functional foods, wellness merchandise and beauty products (Lu, Lou, Hu, Liu, & Chen, 2020).

Ever since Miyazaki and Oikawa (1973), documented the isolation of unrefined GU-0 polysaccharides from the sclerotia of *P. umbellatus*, the interest in the PUPs greatly increased. This growing interest encompasses their preparation (extraction, fractionation, and purification), structural analysis (molecular weight, monosaccharide composition, glycosidic bond types, and structural features), evaluation of biological activity (including structure-activity relationship analysis), the structure-activity relationships (SARs) and its application status. Despite this increase, the research data remains fragmented and scattered across different literature. This review will examine through a rigorous literature review, the different aspect of research on PUPs, so as to provide a valuable reference for future investigations and establish a solid theoretical foundation for further development and application.

## 2 Source

The growth and development of *P. umbellatus* undergoes several stages, including basidiospore germination, mycelial colonization, formation of sclerotia, and maturation of the fruiting body. The sclerotia serve as the primary source for extraction and isolation of various PUPs (He et al., 2017; He, Zhang, Wang, Liang, & Sun, 2015; He, Zhang, Zhang, & Sun, 2016; Li, 2011; Li et al., 2019; Li, Chen, Chen, & Liu, 1995; Li & Wen, 2011; Li, Xu, & Chen, 2010; Liu & Xu, 2007; Miyazaki & Oikawa, 1973; Miyazaki, Oikawa, Yadomae, Yamada, & Ito, 1979; Tan, Guo, & Wang, 2016; Tian, Li, Xu, Jin, & Han, 2005; Ueno, Abe, Yamauchi, & Kato, 1980; Ueno, Okamoto, Yamauchi, & Kato, 1982; Zhang et al., 2015), although studies have also been able to extract the same from the fruiting bodies (Dai et al., 2012; Sun & Zhou, 2014), mycelia (Bi et al., 2013; Kim, Kim, & Kim, 2014; Sun & Zhou, 2014; Tian et al., 2005) and the fermentation liquid (Liu et al., 2019).

In 1973, Miyazaki and Oikawa (1973) reported the extraction of crude polysaccharides GU-0 from the sclerotia of *P. umbellatus*, and the preliminary separation yielded polysaccharides fraction GU-1. Further purification in 1979 (Miyazaki et al., 1979) of GU-0 and GU-1 resulted in the isolation of three highly purified polysaccharides: GU2, GU3, and GU4. Since then, several other polysaccharides have been extracted and characterized from different parts of *P. umbellatus*. From the sclerotia, several PUPs were extracted and separated, which include: the homogeneous *β*-glucan AP (Ueno et al., 1980); the water-soluble polysaccharide WP in 1982, obtained with a yield of 3.9%; the neutral polysaccharide of ZP in 1982, obtained with a yield of 0.15%; ten alkali-soluble polysaccharides AP-1 to AP-10 (Ueno et al., 1982); alkali-soluble glucan P1 and polysaccharide P2 in 1995 (Li et al., 1995); water-soluble polysaccharide PPS2 in 2005 (Tian et al., 2005); heteropolysaccharide PPS in 2010 and 2011 (Li et al., 2010; Li & Wen, 2011); a polysaccharide PUP60S2 in 2015 (He et al., 2015); PPS in 2015 (Zhang et al., 2015); PUP80S1 in 2016, with a yield of 1.08% (He et al., 2016), a heteropolysaccharide PUP60W-1 in 2017, with a yield of 1.16% (He et al., 2017); and heteropolysaccharide PUPS in 2019 (Li et al., 2019). From the fruiting body, a glucan ZPS in 2012 (Dai et al., 2012)and a heteropolysaccharide PUf-C in 2014 (Sun & Zhou, 2014). Isolated and purified from the mycelia: a water-soluble polysaccharide PPS1(Sun & Zhou, 2014) and heteropolysaccharide PUm-C (Tian et al., 2005), in 2005 and 2014 respectively; and two homogenous polysaccharides, GUMP-1-1 and GUMP-1-2, in 2013, with yields of 6.34% and 5.45%, respectively (Bi et al., 2013). From the fermentation liquid of *P. umbellatus*, which contains extracellular and intracellular leach liquor and secondary metabolites as well, in 2019, separated and purified exopolysaccharides PPS1, PPS2, and PPS3 (Liu et al., 2019).

## 3 Preparation technologies of PUPs

The typical process for the extraction, separation, and purification of PUPs divided into four sequential steps as follows: (1) Degreasing – organic solvents are used to eliminate lipophilic impurities such as lipids and pigments, which compromises the purity of the PUPs; (2) Extraction and Concentration – being highly polar, PUPs exhibit different solubility's in both cold water and organic solvents, and thus are transferred from their raw form into hot water, followed by precipitation with ethanol, which reduces their solubility by breaking down the hydrogen bonds between the polysaccharides; (3) Impurity Removal – Co-extracted impurities including proteins, water-soluble pigments, and small polar molecules are eliminated from the crude PUPs; (4) Purification – further purification of PUPs is done using ion exchange chromatography and gel permeation chromatography. These procedures depicting the extraction, separation, and purification of PUPs are illustrated in Figure 2.

### 3.1 Extraction technologies

Hot water extraction (HWE) is one of the traditional methods for PUPs extraction (Bi et al., 2013; Dai et al., 2012; He et al., 2017; He et al., 2016; He et al., 2015; Kim et al., 2014; Li et al., 2019; Li et al., 1995; Li & Wen, 2011; Li et al., 2010; Liu et al., 2019; Liu & Xu, 2007; Lu et al., 2016; Miyazaki & Oikawa, 1973; Miyazaki et al., 1979; Tian et al., 2005; Ueno et al., 1980; Ueno et al., 1982; Zhang et al., 2015). The general process includes grinding the samples to a fine powder, degreasing with ethanol, then dissolving the PUPs with hot water. The mixture is then filtered, concentrated, decoloured, deproteinised, and centrifuged. The supernatant is decanted, then precipitated with ethanol, centrifuged, and lyophilized to obtain crude polysaccharides. To avoid co-precipitation with impurities during ethanol precipitation, it is important to maintain an appropriate concentration of the polysaccharide solution, stir rapidly, and add then slowly ethanol. HWE method has several advantages, including the fact that it needs no special equipment, it's cheap, simple to perform, and suitable for large-scale industrial operations. Its drawbacks are that it has low efficiency, low yield, and has extended extraction durations, is time-consuming and causes partial cleavage of sulfate esters, leading to bioactivity loss.

To overcome these shortcomings, researchers have devised new extraction methods which incorporate other properties of PUPs. These extraction methods include alkaline water solutions (AWE), enzyme-assisted extraction (EAE), microwave-assisted extraction (MAE), and ultrasonic-assisted extraction (UAE).

Polysaccharides containing uronic acid are appropriately extracted using AWE which incorporates alkaline solutions in their protocols, used dilute alkali-solution to extract PUPs (Li et al., 1995; Miyazaki & Oikawa, 1973; Ueno et al., 1980; Ueno et al., 1982). Strong alkalinity hydrolyzes some polysaccharides, and thus the pH must be strictly controlled. Additionally, alkali extracts should also be rapidly neutralized or dialyzed, concentrated, and precipitated using alcohol.

EAE have several advantages such as, they require mild reaction conditions, have high extraction efficiency, protect the environment, and have low energy consumption. The enzymes hydrolyse fungal cell wall, which accelerate the dissolution of PUPs, thus improving extraction efficiency (Zhang et al., 2015). EAE protocols are not without their own drawbacks, for example, the enzymes are sensitive to temperature and pH changes and thus require a lot of optimisations for ideal conditions enzymatic processes to occur. The enzymes being proteinaceous in nature, introduce contaminants in the protocol, further complicating the purification process.

MAE uses the high energy of microwaves to increase the temperature and accelerates the movement molecules which in turn increases the cell pressure and causes the fungal cells to burst the release the PUPs. This improves the efficiency of the extraction protocol (Li, 2011). MAE based protocols are temperature sensitive, and thus require the optimization of microwave power, and pH. MAEs has been found to affect the composition and activity of the polysaccharides.

UAE uses mechanical, cavitation, and thermal effects of ultrasonic waves to destroy cell walls which accelerates the dissolution of PUPs (Bai et al., 2019), thereby improving the efficiency of the extraction protocol. Similar to MAE, the efficiency of UAE, requires optimization of temperature, ultrasonic power, and other factors. UAEs have also been found to affect the composition and activity of polysaccharides (Sun & Zhou, 2014).

PUPs extracted using the above methods (shown in Table 1) undergo fractional precipitation to yield crude PUPs. Fractional precipitation separates PUPs based on their solubility in different concentrations of alcohol. A general rule of thumb is that polysaccharides with low the molecular weights, are less soluble in high concentrations of ethanol, and thus favor their precipitation. He et al., 2017, He, Zhang, Zhang and Sun, 2016 discovered that 60 % ethanol yielded a crude precipitate polysaccharide PUP60, which constituted of two polysaccharide molecules PUP60S2 and PUP60W-1, with had molecular weights of 1.44 × 10^4^ Da and 2.47 × 10^4^ Da respectively. Precipitation with 80 % ethanol yielded PUP80, with the polysaccharide PUP80S1 having a molecular weight of 8.80 × 10^3^ Da. Similalry, the precipitation of the *P. umbellatus* liquid supernatant with ethanol to final concentrations of 50 %, 60 %, and 70 % yielded crude polysaccharides S1, S2, and S3, with molecular weights of 6.90 × 10^4^ Da for PPS1, 4.60 × 10^4^ Da for PPS2, and 3.70 × 10^4^ Da for PPS3 respectively. This further demonstrated that higher ethanol concentrations result in lower molecular weight polysaccharides (Liu et al., 2019).

**Table 1:** Extraction, Separation, Purification of PUPs.

Fraction	Source	Degreasing	Extraction	Separation	Purification	Yields	Refs.
GU-0	Sclerotia	Pre-treated with cold MeOH	Boiling water extraction (1: 5, 2 h × 3)	Dialyzed against running water for 2 days, EtOH precipitation	Not Detected	0.75 %	(Miyazaki & Oikawa, 1973)
GU-1				GU-0 dissolved in Na_2_B_4_O_7_·10H_2_O solution	Cetavlon fractionation	Not Detected	
GU-2	Sclerotia	Pre-treated with cold MeOH	Boiling water extraction (1: 5, 2 h × 3)	GU-1 chromatographed on Sepharose 2 B	Borate-Cetavlon fractionation Sepharose 2B column	Not Detected	(Miyazaki et al., 1979)
GU-3						Not Detected	
GU-4			Hot water (the residue of insoluble 0.4%, Na_2_B_4_O_7_·10H_2_O) extraction, centrifugation (50 °C, 1000 r.p.m.)	EtOH precipitation		Not Detected	
AP	Sclerotia	Pre-treated with cold MeOH	Hot water (1: 10, 9 times) extraction, 10 % aqueous zinc chloride (1: 10, 8 times) extraction, 10 % sodium hydroxide (5% urea, 4 °C, 24 h) Suspending 3 M hydrochloric acid neutralization (4 °C), Dialyzed against running tap-water	Dissolved in 1 % NaOH solution, added dropwise 7 % aqueous copper sulfate precipitation, polysaccharide regenerated	Sepharose CL-2B column with 0.2 M sodium hydroxide	33.9 %	(Ueno et al., 1980)
AP-3					Sepharose CL-2B column with 0.2 M sodium hydroxide	33.9%	
WP			High-pressure extraction (water, 1 kg/cm^2^, 1 h), 2 % sodium hydroxide extraction, water dialysis (4 days)	4 volumes of MeOH precipitation		3.9 %	
ZP			High-pressure extraction (10 % zinc chloride aqueous solution, 1 kg/cm^2^, 1 h), water dialysis (4 days)	4 volumes of MeOH precipitation		0.15%	
AP-1			2 % sodium hydroxide (2 % urea, 4 °C) extraction	HCl neutralization, 4 volumes of MeOH precipitation		6.4%	
AP-2				4 volumes of MeOH precipitation		1.5%	
AP-4			2 % sodium hydroxide (4 °C) extraction	HCl neutralization 4 volumes of MeOH precipitation		1.8%	
AP-5				4 volumes of MeOH precipitation		very small	
AP-6			10 % sodium hydroxide (4 °C) extraction	HCl neutralization 4 volumes of MeOH precipitation		23.8%	
AP-7			2 % sodium hydroxide (2 % urea, 4 °C) extraction	HCl neutralization 4 volumes of MeOH precipitation		2.1 %	
AP-8			2 % sodium hydroxide (2 % urea, 4 °C) extraction	4 volumes of MeOH precipitation	Sepharose CL-2B column with 0.2 M sodium hydroxide	5.0 %	
AP-9			2 % sodium hydroxide (4 °C) extraction	HCl neutralization, 4 volumes of MeOH precipitation		1.7 %	
AP-10			2 % sodium hydroxide (2 % urea, 4 °C) extraction	4 volumes of MeOH precipitation		3.3 %	
P1	Sclerotia	Not Detected	Boiling water extraction	4 volumes of 85 % EtOH precipitation	Sevag method	Not Detected	(Li et al., 1995)
P2			0.1 M NaOH extraction (90 °C, 2 h × 2)	HCl neutralization Dialysis (water, 48 h distilled water, 24 h), 4 volumes of 85 % EtOH precipitation		Not Detected	
PPS1	Mycelia	Not Detected	Water extraction (90 °C, 2 h × 3)	3 volumes of 95 % EtOH (4 °C, overnight) precipitation	Sevag method, dialyzed against (distilled water, 72 h), 3 volumes of 95 % EtOH precipitation	Not Detected	(Tian et al., 2005)
PPS2	Sclerotia						
PPS	Sclerotia	Not Detected	Boiling water extraction (1: 8, 1: 6, 1: 6, 2 h × 3)	95 % EtOH (80 %, v/v) precipitation	Sevag method, H_2_O_2_ depigmentation, dialyzed against (water 72 h, distilled water, 12 h), EtOH (70 %, *v*/v，overnight) Precipitation	0.369 %.	(Liu & Xu, 2007)
PPS	Sclerotia	Not Detected	Boiling water (1: 10, 1 h) extraction	4 volume of 95 % EtOH (4 °C, overnight) precipitation, dialyzed against (distilled water, 7 days)	Sevag method, 0.22 um filter 5 kDa PM5 membrane, DEAE-cellulose column with 2 M NaCl, Sepharose-6B column (2.5 × 95 cm) with 0.15 M NaCl	Not Detected	(Li et al., 2010; Li & Wen, 2011)
PUP80S1	Sclerotia	Soaked overnight (95 % EtOH) Defatting for 2 times	Boiling water (2 h × 3) extraction	95 % EtOH (80 %, *v*/v) precipitation	DEAE Sepharose Fast-Flow column (2.6 × 100 cm) with a step-wise gradient of 0, 0.1, 0.2 and 0.4 M NaCl, Sephacryl S-100/200 column (1.6 × 100 cm) with water	1.08%	(He et al., 2016)
ZPS	Fruiting body	Pre-treatment with EtOH (1: 3, 95 %, 100 °C, 1.5 h)	Distilled Water extraction (1: 3, 100 °C, 1.5 h × 3)	EtOH (95 %, 1: 4 *v*/v, 4 °C, 12 h) precipitation	Remove proteins (distilled water), DEAE-52 cellulose column (5.0 × 70.0 cm) with a 10-step gradient of 0–2 M NaCl, Sepharose CL-6B column (2.6 × 100 cm) with water	Not Detected	(Dai et al., 2012)
PUm-C	Mycelia	Not Detected	Ultrasonic extraction (1: 15，500 W, 18 kHz, 25 min), equal volume of hot water (98 °C, 1.5 h) extraction	EtOH (80 %, *v*/v) precipitation	Sephadex G-100 column	Not Detected	(Sun & Zhou, 2014)
PUf-C	Fruiting Body					Not Detected	
GUMP-1-1, GUMP-1-2	Mycelia	Pre-treatment with EtOH (1: 5, 80 %, 80 °C, 24 h)	Hot water (90 °C, 4 h × 2) extraction	Not Detected	Sevag method, dialysis (Cut-off Mw 3500 Da), DEAE-52 Cellulose column with 0, 0.2, 0.4 and 0.6 M NaCl, Sephacryl S-400 column (2 × 60 cm)	6.34% and 5.45 %.	(Bi et al., 2013)
PPS	Sclerotia	Not Detected	Reflux extraction with water (1: 10 g/mL, 100 °C, 30 min), centrifugation, filtration, concentration, spray drying	Not Detected	Not Detected	Crude yields 5 %; 8.69 %.	(Zhang, et al., 2015)
Khz-cp	Mycelia	Not Detected	High-pressure extraction (1: 8, 115 °C, 1 h × 2)	Sevag method 4 volumes of 95 % EtOH precipitation	Dialysis (cut-off Mw100000 Da)	Not Detected	(Kim et al., 2014)
PUP60S2	Sclerotia	Soaked with 95 % EtOH overnight defatting for 2 times	Boiling water (2 h × 3) extraction	95 % EtOH (60 %, *v*/v) precipitation	DEAE Sepharose FF column (2.6 × 100 cm) with a step-wise gradient of 0, 0.1, 0.2 and 0.4 M NaCl, Sephacryl S-100/300 column (2.6 × 100 cm) with 0.2 M NaCl	Not Detected	(He et al., 2015)
PPS	Sclerotia	Not Detected	Microwave-assisted extraction (l:25 g/mL, 75 °C, PH 6.5, 400 W, 3 min)	Not Detected	Sevag method	2.84 %	(Li, 2011)
PUP60W-1, PPUS	Sclerotia	Soaked overnight (95 % EtOH) Defatting for 2 times	Boiling water (2 h × 3) extraction	95 % EtOH (60 %, *v*/v) precipitation	DEAE Sepharose FF column (2.6 × 100 cm) with distilled water, Sephacryl S-200 High-Resolution column (2.6 × 100 cm) with distilled water	1.16%	(He et al., 2017; Li et al., 2019)
PB	Sclerotia	Degreased with petroleum ether, ethyl acetate, and ethanol	Warm water maceration (1: 200, 1 h × 2)	Microcrystalline cellulose precipitation	Separated by different molecular weight membranes (MWCO200、400、800、5000、10,000、50,000、100,000)	Not Detected	(Lu et al., 2016)
PPS1, PPS2, PPS3	Fermentation Liquid	Not Detected	Water immersion (grown under dynamic conditions in a 50 L liquid medium, 25 °C, 6 days)	95 % EtOH (50 %, *v*/v; 60 %, v/v; 80 %, *v*/v) precipitation	Sephadex G-100 column with eluting with deionized water	Not Detected	(Liu et al., 2019)
HPP	Not Detected	Not Detected	Not Detected	Not Detected	Sevag method, DEAE-52 cellulose column (3 × 35 cm) and eluted with deionized water, Sephadex G-100 column with deionized water	Not Detected	(Jia et al., 2021; Liu et al., 2020)
PUP-W-1	Sclerotia	Pre-treatment with 95 % EtOH (1: 10 *v*/v, 90 °C, 2 h, twice)	boiling water extraction (1: 12 *v*/v, 2 h × 2) purification	95 % EtOH (80 % v/v, room temperature, 12 h) precipitation	DEAE-Sepharose FF column and eluted with ddH_2_O and NaCl (0.1, 0.2, 0.5, and 1.0 mol/L), Superdex ™ G-75 column with 0.2 M NaCl	0.112 %	(Gao et al., 2023)

### 3.2 Separation and purification technologies

Prior to the separation of PUPs, various steps are taken to facilitate the separation operation. Since proteins, similar to polysaccharides, are polar macromolecules, must be removed from the mixture using the Sevag method for deproteinizing polysaccharides. The Sevag method has been successfully employed by researchers for the deproteinization of PUPs (Li et al., 1995; Li et al., 2010; Tian et al., 2005; Bi et al., 2013; Jia et al., 2021; Kim et al., 2014; Li & Wen, 2011; Liu et al., 2020; Liu, Bai, & Gong, 2013). The Sevag method has widespread use, it owing to its mild reaction conditions. However, it has low efficiency and leaves behind a significant amount of toxic chemicals. Future studies need to devise new protocols which are more efficient and environmentally friendly. For example, resin adsorption and enzyme hydrolysis and proper candidates for deproteinization of PUPs.

In addition to protein contaminants, pigments should also be removed from the crude extract of PUPs. Hydrogen peroxide (H_2_O_2_) was employed for decolorization of the crude PUPs solution (Liu & Xu, 2007). Using 3 % dilute ammonia solution (NH_4_OH), the pH was first adjusted to between 8 and 9, and then while stirring 30 % H_2_O_2_ is added to a 3:1 ratio. The mixture was then incubated at 50 °C for 2.5 h. The resulting solution was precipitated using 95 % ethanol (80 %, *v*/v), followed by dialysis in running water for 72 h and finally distilled water for an additional 12 h. The dialysate was then precipitated with absolute ethanol (70 %, v/v), and subjected to freeze-drying to obtain purified PUPs. The polyamide resin treatment is an equally effective method for discolourisation, which offers high stability, selective adsorption, and reusability. Polyamide resin was utilized to discolourise the crude PUP concentrate (Liu et al., 2013). This was done by first adding 95 % ethanol (85 %, *v*/v) to attain a relative density of 1.10 (20 °C). The resulting mixture was collected, precipitated, and then dissolved to form an aqueous solution with a density of 1 g/mL. The liquid was then discolourised using a polyamide resin at a ratio of 1:10. The resulting effluent was collected and concentrated to obtain a discoloured crude polysaccharide concentrate with a relative density of 1.15 (20 °C).

The separation and purification of crude PUPs are typically done using chromatographic columns, mainly ion exchange and gel columns. Anionic ion-exchange chromatography is generally used to separating the crude PUPs, with the purified product eluted using distilled water or NaCl solution. The separation process of anionic ion-exchange chromatography involves the adsorption-desorption of acidic PUPs through ion exchange. The negatively charged acidic PUPs are selectively adsorbed onto the anion-exchange cellulose, while neutral ones smoothly pass through. This makes it suitable for separating various types of acidic PUPs, neutral polysaccharides, and mucopolysaccharides. A linear NaCl gradient can also be used to elute the adsorbed acidic polysaccharides from the column. While DEAE-cellulose column (Bi et al., 2013; Dai et al., 2012; Jia et al., 2021; Li & Wen, 2011; Liu et al., 2020) and DEAE Sepharose FF column (He et al., 2015; He et al., 2016; He et al., 2017; Li et al., 2019) were used to separate crude PUPs. Gel column chromatography, also known as molecular sieving, is a technique for separating polysaccharides based on their size and shape. They include: Sepharose 2B was utilized for purification of GU-1 (Miyazaki et al., 1979); Sepharose CL-2B was employed for purification of PUPs (Ueno et al., 1980; Ueno et al., 1982); Sephacryl S series were used to further purified PUPs to obtain homogeneous polysaccharides (Bi et al., 2013; He et al., 2015; He et al., 2016; He et al., 2017; Li et al., 2019); Sepharose CL-6B was used for purification of ZPS (Dai et al., 2012); Sepharose 6B was utilized for purification of PPS (Li & Wen, 2011; Li et al., 2010; Liu & Xu, 2007); Sephadex G series were employed for purifying PUPs (Jia et al., 2021; Liu et al., 2019; Liu et al., 2020; Sun & Zhou, 2014). Elution is usually performed using NaCl and distilled water solutions. Specific separation and purification methods are also summarized in Table 1.

Polysaccharides are biopolymers formed by the polymerization of single or multiple monosaccharide monomers. This structural diversity, encompassing variations in molecular weight, monosaccharide composition, branching patterns, glycosidic bond linkages, and other chemical features, directly influences the diverse biological functions exhibited by these macromolecules. Several PUPs with distinct chemical structures and functionalities have been reported. The observed variability in the biological activities of PUPs can be attributed to several factors, including the diverse origins of the source material and variations in isolation, extraction, and purification methodologies. Subtle differences in the chemical structure of these polysaccharides, influenced by these factors, significantly impact their biological functions. Therefore, a comprehensive characterization of their chemical structures is crucial for understanding and predicting their biological activities.

## 4 Chemical characterization of PUPs

Polysaccharides are macromolecular compounds formed by the polymerization of single or multiple monosaccharides. Their structural characteristics determine their biological activity. The biological functions of polysaccharides may vary depending on factors such as molecular weight, monosaccharide composition, degree of branching, glycosidic bond type, and other chemical structural features. There exists a multitude of PUPs with distinct chemical structures and functionalities. This is owing to the diverse origins of *P. umbellatus* and variations in isolation, extraction, and purification methods of the PUPs. Small differences in their structure affect their specific biological functions and thus a comprehensive description of their chemical structure is essential for determining their biological activities.

### 4.1 Determination of molecular weights

Various chromatographic techniques have been employed to determine the weight-average molecular weight (Mw) of PUPs, including size exclusion chromatography (SEC), high-performance gel permeation chromatography (HPGPC) coupled with evaporative light scattering detection (ELSD) or refractive index detection (RID), and SEC coupled with multi-angle laser light scattering (MALLS) and RID. The reported Mw of PUPs range from 6.88 × 10^3^ to 2.27 × 10^6^ Da, as determined by the methods mentioned above are summarized in Table 2.

**Table 2:** Molecular weight determination of PUPs.

PUPs	Mw (Da)	Chromatographic method				Refs.
		Column	Mobile phase	Standard	Detection	
AP-3	1.20 × 10^6^	Sepharose CL-2B	0.2 M NaOH	Dextran	Phenol‑sulfuric acid method	(Ueno et al., 1980)
PPS	1.60 × 10^5^	TSK-GEL G600 PWXL TSK-GEL G4000 PWXL	Water and 0.5 M NaCl	Not Detected	RID-MALLS	(Liu & Xu, 2007)
ZPS	2.27 × 10^6^	TSK-GEL G4000 PWXL /Ultra-hydrogel linear	Water	Dextran	HPLC-ELSD /SEC-ELSD	(Dai et al., 2012)
GUMP-1-1	2.50 × 10^5^	TSK-GEL G3000 SWXL	0.1 M Na_2_SO_4_	Dextran	HPGPC-RID	(Bi et al., 2013)
GUMP-1-2	5.0 × 10^4^					
PUm-C	8.57 × 10^5^	NOT DETECTED	3 M NaAc	Dextran	HPLC-RID	(Sun & Zhou, 2014)
PUf-C	6.79 × 10^5^					
PUP60S2	1.44 × 10^4^	TSK-Gel G4000 PWXL	0.1 M NaNO_3_	Dextran	HPSEC-RID	(He et al., 2015)
PUP80S1	8.80 × 10^3^	TSK-Gel G4000 PWXL	0.1 M NaNO_3_	Dextran	HPSEC-RID	(He et al., 2016)
PUP60W-1	2.47 × 10^4^	TSK-Gel G5000 PWXL TSK-Gel G3000 PWXL	Water	Not Detected	RID-MALLS	(He et al., 2017)
PPS1	6.90 × 10^4^	Ultra-hydrogel 250	0.1 M NaAc	Dextran	HPGPC-RID	(Liu et al., 2019)
PPS2	4.60 × 10^4^					
PPS3	3.70 × 10^4^					
HPP	6.88 × 10^3^	TSK gel G4000 PWXL	Water	Dextran	HPGPC-RID	(Jia et al., 2021; Liu et al., 2020)

Regardless of the method used to determine the Mw of PUPs, the separation mechanism of SEC, HPGPC-ELSD and HPGPC-RID is similar. Larger molecules are prevented from penetrate the pores within the stationary phase, resulting in their faster elution, while the smaller molecules permeate through the pores where relative to their sizes, they experience different levels of retardation as they move through the gel column. Using pullulan or dextran standards, calibration curves are generated to calculate the Mw of polysaccharides based on elution volume. For example, the Mw of an alkali-soluble polysaccharide AP extracted from sclerotia, measured using SEC on a Sepharose CL-2B column with dextran standards, was determined to be ∼1.2 × 10^6^ Da based on the elution volume detected using the phenol‑sulfuric acid colorimetric analysis (Ueno et al., 1980). HPGPC coupled with ELSD or RID is commonly used to determine molecular weights of PUPs due to its speediness and reliability. HPGPC-ELSD was used with water as the mobile phase for determining the Mw of ZPS, which was determined to be 2.27 × 10^6^ Da (Dai et al., 2012). Gel-Filtration Chromatography (GPC) works by minimizing or avoiding nonspecific adsorption between polysaccharides and gels. This can be achieved using a non-volatile buffer salt with a specific ionic strength as the mobile phase. Detection using ELSD requires a volatile mobile phase, which limits the use of non-volatile buffer salts. Because of its compatibility with various mobile phases HPGPC-RID has become the main technique for determining Mw of PUPs. The Mw values of GUMP-1-1 and GUMP-1-2 were determined to be 2.50 × 10^5^ Da and 5.0 × 10^4^ Da respectively (Bi et al., 2013), while those of PPS1, PPS2, and PPS3 were 6.90 × 10^4^ Da, 4.60 × 10^4^ Da, 3.70 × 10^4^ Da respectively (Liu et al., 2019). The Mw of HPP was determined to be 6.88 × 10^3^ Da (Jia et al., 2021; Liu et al., 2020) when measured using HPGPC-RID with dextran standards.

The precision of HPGPC is inherently variable owing to the disparity in hydrodynamic volume which results from the diverse structural characteristics between PUPs and standards. Over the years, SEC-MALLS-RID has gained prominence for the determination of the Mw of PUPs owing to its high accuracy, reproducibility, and independence from reference standards. For example, using SEC-MALLS-RID (He et al., 2017; Liu & Xu, 2007) were able to quantify the Mw of PPS and PUP60W-1 as 1.6 × 10^5^ Da and 2.47 × 10^4^ Da with polydispersity indices, (Mw/Mn, where Mn is number average molecular weight) of 2.914 and 1.04, respectively.

### 4.2 Determination of constituent monosaccharides

Accurately determining the monosaccharide composition of polysaccharides is essential for understanding the structure of these carbohydrates. The analysis basically involves a complete acid hydrolysis of polysaccharides to dissociate into the composite monosaccharides, which are then taken for further examination. To determine the monosaccharide composition of PUPs, various techniques are used, including, paper chromatography, thin-layer chromatography (TLC), gas chromatography (GC) coupled with flame ionization detection (FID) or mass spectrometry (MS) using pre-column acetylation derivatisation, and high-performance liquid chromatography-ultraviolet (HPLC-UV) with pre-column 1-phenyl-3-methyl-5-pyrazolone (PMP) derivatisation. The constituent monosaccharides in PUPs include glucose (Glc), galactose (Gal), mannose (Man), fructose (Fru), xylose (Xyl), fucose (Fuc), galacturonic acid (GalA), and glucuronic acid (GlcA). The hydrolysis and analysis methods used to determine the monosaccharide composition of PUPs are summarized in Table 3.

After acid hydrolysis (1 MN H_2_SO_4_, 100 °C, 5 h), the monosaccharide composition of GU-0 using paper chromatography, and identified Glc as the major component along with trace amounts of Gal, Man, Xyl, and GlcA. Similarly, after acid hydrolysis (1 N H_2_SO_4_, 100 °C, 5 h) GU-1 was found to be composed of mainly of Glc trace amounts of GlcA (Miyazaki & Oikawa, 1973). Ueno et al. (1982) also measured the monosaccharide composition of PUPs, specifically the water-soluble WP, the neutral polysaccharide ZP, and the alkali-soluble polysaccharides AP-1 to AP-10, using paper chromatography, after an acid hydrolysis (0.5 M sulfuric acid overnight at 100 °C) step. WP was found to be composed of mainly composed D-Glc and trace amounts of D-GlcA and D-Man, ZP of D-Glc and D-Man., while AP-1 to AP-10 were exclusively of Glc.

In 1980, AP was conducted gas-liquid chromatography (GLC) analysis of AP after an acid hydrolysis (1 M HCl in methanol, 100 °C, 24 h) step, and subsequent trimethylsilylation with hexamethyl-disilazane (HMDS) and trimethylchlorosilane (TMCS) in pyridine (TMS-HT). The results revealed that AP was exclusively composed of Glc (Ueno et al., 1980). In the past few years, GC or HPLC have been used more frequently in the studies analyzing the monosaccharide composition of PUPs. The GC analyses conducted revealed that the monosaccharide composition of PPS consisted mainly of D-Glc with trace amounts of D-Man and D-Gal (Li et al., 2019). PUP60S2 was found to be mainly composed of D-Glc and ∼ 22.3 % GlcA (He et al., 2015); PUP60W-1 was composed of Glc, Fuc, and Gal in molar ratios of 0.9:1:13.3 respectively (He et al., 2017); PUP80S1 was mainly composed Glc and ∼ 8.5 % GlcA (He et al., 2016); GUMP-1-1 was composed of Man, Glc, Fru, and Gal in molar ratios of 1.4:17.4:0.4:1.2 respectively; GUMP-1-2 comprises Man, Glc, Fru, GlcA, and Gal as constituent monosaccharides in the molar ratios of 1.2: 11.4: 0.2: 2.1: 1.1 respectively (Bi et al., 2013); ZPS was predominantly composed (>90 %) of Glc; while PUPS was composed of Gal, Fuc, and Glc in molar ratios of 13.24:1.03:0.88 respectively (Dai et al., 2012); HPP was exclusively composed of Glc (Liu et al., 2020).

HPLC was employed to assess the monosaccharide composition of PUPs (Liu et al., 2020; Liu et al., 2019; Sun & Zhou, 2014; Zhang et al., 2015). His study revealed that the fruiting body polysaccharide PUf-C and the mycelial polysaccharide PUm—C, to be composed of Gal to Glc in molar ratios of 1:5.42, and 1:1.57 respectively. In analyzing the fermentation liquid as a unique PUP source, the composition of the extracellular polysaccharides PPS1, PPS2, and PPS3 had varying molar ratios of Man, Gal, and Glc. Song, Nan, Yuan, Jin, and Hu (2019) optimized ultra-performance liquid chromatography in tandem with mass spectrometry (UPLC-MS) to determine the monosaccharide composition of *P. umbellatus* from 17 different parts which consistently revealed on PUPs from all 17 sources, being composed of eight monosaccharides (Glc, Fuc, Gal, Rib, Xyl, Ara, Man, and GlcA). Rha, Fru, and GalA were also detected in multiple PUPs, thus aligning with the reported monosaccharide profiles.

### 4.3 Determination of glycosidic linkages

Methylation analysis and/or periodate oxidation are the main methods used to determine the linkage patterns between monosaccharide residues, thus enabling the quantitative analysis of sugar residues. The Hakomori method (AP (Ueno et al., 1980), GU-1 (Miyazaki & Oikawa, 1973)), Needs (ZPS (Dai et al., 2012)) method, Ciucanu method (PUP60S2 (He et al., 2015)) and Anumula method (PUP80S1 (He et al., 2016)) are regularly used for polysaccharide methylation. Complete methylation, which is essential for methylation analysis, can be verified by FT-IR analysis. The disappearance of the hydroxyl stretching vibration peak within the 3600–3200 cm^−1^ range, indicates a successful and complete methylation of the polysaccharides.

While the use of methylation derivatisation proves to be beneficial for glycosidic linkage analysis, it does not provide information on the sequential arrangement of constituent monosaccharides or the anomeric configuration of glycosidic linkages. In it, fully methylated polysaccharides undergo through methanolysis and acid hydrolysis, followed by TLC, paper electrophoresis, and GLC. For example, Miyazaki and Oikawa (1973) first methylated GU-1 using DMSO-NaH-CH_3_I. The methylated product was then subjected to methanolysis with 0.5 N MeOH-HCl, and placed in a boiling water bath for 10 h. GLC analysis of the methylated GU-1 revealed four peaks which corresponded to the alditol from terminal-Glc*p*; 1,6-linked-Glc*p*; 1,4-linked-Glc*p*; 1,3-linked-Glc*p.* Hydrolysis of the methylated GU-1 using 90 % HCOOH and 1 N H_2_SO_4_, resulted in products that were separated by TLC and identified by paper electrophoresis, which indicated that GU-1 possessed 1,6-*β*-D-Glc*p*, 1,4-*β*-D-Glc*p*, and 1,3-*β*-D-Glc*p* linkages with branching at the C-3 or C-6 position of the glucose residues. Ueno et al. (1980) used a similar method to methylate AP, after which the resultant product was hydrolyzed with 90 % formic acid for 2 h at 100 °C, followed by 12 h of hydrolysis with 0.25 M sulfuric acid at 100 °C aslo. GLC analysis of the alditol acetates revealed peaks corresponding to terminal-Glc*p,* 1,3-linked-Glc*p*, 1,3,6-linked-Glc*p* with the molar ratios being 1:2.4:0.9. Further analysis of the methylation products with GLC-MS, indicated that AP had a backbone of 1,3-*β*-D-Glc*p*, branching at the C-6 position, and a single D-Glc group at every third D-Glc*p* residue.

Fully methylated polysaccharides can also be hydrolyzed using acid, to yield a hydrolyzed product that can be further converted into partially methylated alditol acetates (PMAA) for analysis using gas chromatography-mass spectrometer (GC–MS). Glycosidic linkage patterns are analysed, by subjecting PUPs to sequential methylation, hydrolysis, reduction, and acetylation, resulting in the formation of PMAA. followed by quantitative and qualitative analysis using GC–MS. The backbone and branch structures of PUPs, along with their analyses, are summarized in Table 4.

HPLC was employed to assess the monosaccharide composition of PUPs (Liu et al., 2020; Liu et al., 2019; Sun & Zhou, 2014; Zhang et al., 2015). PMAA follows a specific cleavage pattern during electron dissociation and is identified based on mass spectrometric fragments and retention times. The resulting PMAA and monosaccharide compositions are used to determine the linkage pattern sof sugar residues in the polysaccharides. ZPS was utilized the Needs method for methylation (Dai et al., 2012), which resulted in five main PMAAs corresponding to the different glycosidic linkage forms, including 1,6-linked-Glc*p*, 1,3,6-linked-Glc*p*, 1,4-linked-Glc*p*, 1,3-linked-Glc*p*, and non-reducing terminal-Glc*p*. Combined with 1D and 2D NMR spectra analysis, ZPS was ditermined to have a structure with a 1,4-*β*-D-Glc*p* and 1,6-*β*-D-Glc*p* backbone, substituted at the C-3 position of 1,6-*β*-D-Glc*p* by 1,3-*β*-D-Glc*p* branches.

Using the Ciucanu and Caprita method, PUP60S2 methylated revealing five glycosidic linkage forms, including: terminal-Glc*p*, 3-linked-Glc*p*, 4-linked-Glc*p*, 6-linked-Glc*p* and 3,6-linked-Glc*p* with their respective molar ratios. Combined with 1D and 2D NMR spectra analysis, PUP60S2 was determined to consist of 1,6-*β*-D-Glc*p*, with every second residue being substituted at position C-3 by side chains consisting of various glucose units (He et al., 2015). Using the Anumula and Taylor method combined with 1D and 2D NMR spectra analysis, PUP80S1 was analysed and found it to have a 1,6-*β*-D-Glc*p* and 1,3-*β*-D-Glc*p* backbone (He et al., 2016).

PUP60W-1 was used Anumula & Taylor method to reveal the presence of various substituted sugar units, including: 1-linked-Gal*p*, 1-linked-Fuc*p*, 1,2-linked-Gal*p*, 1,3-linked-Glc*p*, 1,4-linked-Glc*p*, 1,6-linked-Glc*p*, 1,2,3-linked-Gal*p*, and 1,2,6-linked-Gal*p*, with galactose being the predominant component. Further analysis with 1D and 2D NMR, indicated that PUP60W-1 had a 1,6-*α*-D-Gal*p* backbone, with branches at the C-2 position, where two out of every three main chain galactosyl residues replacing terminal galactosyl residues (He et al., 2017). In addition to methylation and GC–MS analysis, Periodate oxidation and Smith degradation are used to determine the glycosidic linkage pattern of PUPs.

Periodate oxidation selectively breaks adjacent dihydroxy or trihydroxy groups in sugar molecules to produce the corresponding polysaccharide aldehydes, formaldehyde, or formic acid. The processes of determining the position of glycosidic bond, the degree of polymerization in linear polysaccharides, and branching patterns in branched polysaccharides, involves the quantification of formic acid release and periodate consumption.

Smith degradation involves the acid hydrolysis of the periodate oxidation products after reduction and identifying the hydrolysates, so as to infer the positions and branching points of glycosidic bonds. For example, GU-1 consumed 1.09 mol of periodate and produced 0.39 mol of formic acid per glucose unit through periodate oxidation. The molar ratio of degradation products (glucose, erythritol, and glycerol) was 1: 0.47: 0.90, suggested that GU-1 primarily contained linkages at C-3 or C-6 positions, with branches (Miyazaki & Oikawa, 1973). AP (Ueno et al., 1980) was reported to consume 0.45 mol of periodate per unit of Glc and release 0.24 mol of formic acid, as calculated by the Fleury Lange method. AP-1 was then completely hydrolyzed with an acid, and GLC used to determine the presence of Glc and glycerol as hydrolysis products with the molar ratio of 1:2.4:0.9 based on subpeak area. The structure of the compound was characterized using GLC-MS, confirmed that AP mainly contained 1,3-linked-Glc*p* with branches at C-6 sites of 1,3-*β-*D-Glc*p* residues.

Dai et al. (2012) selectively broke the 1,4-linked-Glc*p,* 1,6-linked-Glc*p* bonds using high acid oxidation and NaBH_4_ reduction reactions. The ZPS side chain was further characterized by LC-MS analysis, which revealed its main chain to be 1,6-*β*-D-Glc*p*, branched at the C-3 position. With the length of the connected side chain 1,3-*β*-D-Glc*p* chain consisting of 1, 2, or 3 residues. The possible structure of PUPs are illustrated in Fig. 3.

## 5 Biological activities of PUPs

PUPs are the primary active constituents in *P. umbellatus*. They exhibit diverse biological activities, including immunomodulatory, antitumor, antioxidant, and other pharmacological effects, due to their diverse and complex structures.

### 5.1 Immunomodulation activity

**Fig. 4:**
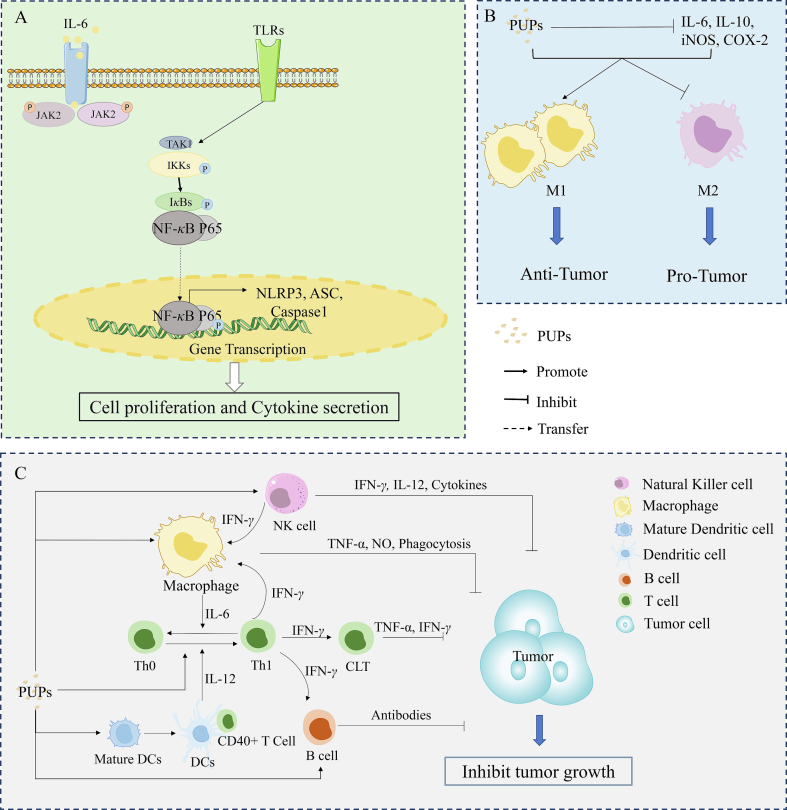
Immunomodulatory mechanisms of PUPs. A: PUPs activate intracellular signaling pathways *via* a variety of surface receptor binding modes (TLRs), and finally promote the proliferation and activation of immune cells. B, C: Various immune cells interact with each other to form an immune regulation network, resulting in inhibiting the growth and metastasis of tumor and PUPs reduce the concentration of TNF-*α,* NO, so as to promote the differentiation of M1 macrophages, and activate DC cells, allowing them to function normally in antigen presentation., which plays an anti-tumor role.

Immunomodulation activity is a prominent biological function of natural polysaccharides (Guo et al., 2022; Zhu et al., 2016). Polysaccharides possess various immunomodulatory activities, such as activating dendritic cells (DCs) and macrophages, promoting splenocyte proliferation, and enhancing the function of the host immune system. The key aspect of the immunomodulatory effect of PUPs is their cellular immune function (Gong et al., 2020). The immunomodulatory mechanisms of PUPs are displayed in Fig. 4.

PPS was extracted from *P. umbellatus* sclerotia was treated to mouse bone-derived dendritic cells (BMDCs), and the results revealed a dose-dependent upregulation of interleukin-12 (IL-12) p40, IL-10, and cluster of differentiation 86 (CD86) expression on the cell surface of BMDCs. Furthermore, PPS treatment enhanced T-cell activation while reducing phagocytosis in BMDCs. In addition, combined flow cytometry (FCM) analysis showed that monoclonal antibodies targeting Toll-like receptor 4 (TLR4) inhibited PPS-induced production of IL-12 p40. Together, these findings suggest that PPS promotes the activation and maturation of BMDCs *via* the TLR4 pathway, thereby exerting an immunomodulatory effect in mice (Li et al., 2010). PPS extracted from *P. umbellatus* sclerotia significantly stimulated the proliferation of splenocytes and peritoneal macrophages (pMφs), increasing the production of inflammatory mediators (nitric oxide (NO)) and cytokines (IL-1*β* and tumor necrosis factor-alpha (TNF-*α*)) in C3H/HeN mice. The results of combined FCM and confocal laser-scanning microscopy analyses showed that PPS promoted the activation and maturation of macrophages *via* the TLR4-modulated nuclear factor kappa B (NF-*κ*B) pathway (Li et al., 2011). ZPS exhibited a potent immunomodulatory effect by stimulating IL-1*β* production in peritoneal macrophages and promoting the proliferation of splenic B cells in mice (Dai et al., 2012). GUMP-1-1 and GUMP-1-2, isolated from *P. umbellatus* mycelia, demonstrated antitumor activity by enhancing immune response (Bi et al., 2013). These compounds significantly suppressed H22 tumor growth in BALB/c mice and extended the survival time of H22 tumor-bearing mice. They also remarkably increased spleen weights and splenocyte proliferation in H22 tumor-bearing mice. Water-soluble heteropolysaccharides PPS1, PPS2, and PPS3 extracted from *P. umbellatus* fermentation liquid possess strong immune cell activation effects, activating macrophages and increasing phagocytosis *in vitro*. For example, the effect of 100 μg/mL PPS1 concentration may reach 8.0 μM of macrophage NO production, which is ten times more than the control (0.8 μM) (Liu et al., 2019). In the bladder cancer microenvironment *in vitro*, 10 μg/mL of HPP significantly increased the secretion of multiple interferon-gamma (IFN-γ)-stimulated RAW264.7 immune factors, such as IL-1*β*, IL-6, IL-23, NO, regulated upon activation, normal T cell expressed and secreted (RANTES), and the expression of M1 phenotype indicator CD80. FCM demonstrated that HPP was recognized by TLR2 and activated NF-*κ*B and nucleotide-binding domain, leucine-rich-containing family, pyrin domain–containing-3 (NLRP3) signaling pathways, thereby enhancing the host immune system and exerting anti-tumor effects (Liu et al., 2020).

Additionally, PUPs have been employed *in vivo* to ameliorate the adverse effects associated with Bacille Calmette-Guerin vaccine (BCG) treatment, thereby garnering increasing attention toward minimizing the side effects of BCG while preserving its efficacy. An N-butyl-N-(4-hydroxybutyl) nitrosamine (BBN)-induced bladder cancer model was established to assess the impact of PPS combined with BCG on bladder cancer in rats. The findings demonstrated that the combination of PPS administration and intravesical BCG injection significantly increased bladder weight while fully reversing the adverse effects associated with single BCG treatment in the short term. FCM analysis results also revealed that the expression of costimulatory molecules D86, D40, and TLR4/D14 was upregulated after the combined application of BCG and PPS. Changes in the spleen and thymus suggested that PPS possesses a potent immunoprotective effect (Zhang et al., 2015).

### 5.2 Antitumor activity

The antitumor effects of PUPs were first identified in mouse S-180 sarcoma cells in 1973 (Ito, Naruse, & Sugiura, 1976). The extracted GU-2, GU-3, and GU-4 were administered via intraperitoneal injection (1 or 5 mg/kg/day, for 10 days) to act on mouse S-180 sarcoma cells (subcutaneously implanted, 30 mg). It was found that GU-3, containing (1 → 3)-*β* linkages and branching at C-6, exhibited strong antitumor activity (5 mg/kg/day, tumor regression at 42 days, 86.2 %) (Miyazaki et al., 1979). Ueno, Abe, Yamauchi and Kato, 1980, Ueno, Okamoto, Yamauchi and Kato, 1982 investigated the inhibitory activity of the water-soluble polysaccharide WP and the alkali-soluble polysaccharide AP and its derivatives (intraperitoneal injection, 10 mg/kg/day, for 10 days) on mouse S-180 sarcoma cells. The findings revealed that the inhibition rate of WP was 100 % and that of AP and its derivatives was 57–100 % in the 5th week. The inhibitory activity of the water-soluble polysaccharide P1 and the alkali-soluble polysaccharide P2 (intraperitoneal injection, 1 mg/kg/day, for 10 days) on mouse S-180 sarcoma cells and found that the inhibitory rates of P1 and P2 were 80.2 % and 16.1 %, respectively. Meanwhile, the tumor inhibition rate of P1 and P2 increased to 87.4 % and 24.2 %, respectively, after removing the side chain (Li et al., 1995).

The antitumor activity of PUPs may be associated with the stimulation of host immunomodulatory mechanisms, thereby exerting an anti-tumor effect through augmentation of the host immune system function. To date, remarkable progress has been made in tumor immunotherapy (Dai, Zhao, Li, Yu, & Gong, 2020; Drake & Stein, 2018; Zhou et al., 2024). PPS exerts anti-tumor effects in the bladder cancer microenvironment by interfering with TLR-4, nitric oxide synthase (iNOS), and cyclooxygenase (COX)-2-regulated NF-*κ*B (P65) signaling and stimulating macrophage polarization toward the M1 phenotype. It selectively binds to specific surface receptors, CD86 and CD40, thereby eliciting an immunomodulatory response in macrophages (Liu, Zhang, Tan, Xu, & Zeng, 2017). Bi et al. (2013) established a mouse liver cancer H22 transplantation model by subcutaneously injecting H22 cells into BALB/c mice. After inoculation of H22 cells, mice were given GUMP-1-1 and GUMP-1-2 orally (100 and 200 mg/kg) once a day for 10 days. Results showed that both GUMP-1-1 and GUMP-1-2 significantly increased spleen weight and splenic cell proliferation in H22 tumor-bearing mice, playing an antitumor role by improving immune response. Notably, treatment with both compounds resulted in a substantial reduction in tumor volume in H22 tumor-bearing mice, with inhibitory effects observed for GUMP-1-1 (200 mg/kg, 45.2 %) and GUMP-1-2 (200 mg/kg, 63.2 %). Furthermore, administration of either compound prolonged the survival time in H22 tumor-bearing mice, with survival rates of 56.3 % and 85.8 % for GUMP-1-1 (200 mg/kg) and GUMP-1-2 (200 mg/kg), respectively. RAW 264.7 cells were exposed to T24 cell culture supernatant to replicate the bladder tumor microenvironment (TME) in an *in vitro* setting. HPP exhibited significant immune activity and increased secretion of various IFN-γ-stimulated immune factors, such as IL-1*β*, IL-6, IL-23, NO, and RANTES in macrophages in the TME model, and expression of the M1 phenotype indicator CD80. HPP also recognized and activated NF-*κ*B and NLRP3 signaling pathways *via* TLR2, exerting anti-tumor effects by enhancing host immune system function (Liu et al., 2020).

PB enhanced Caspase-3 expression by downregulating protein kinase B (AKT) overexpression, which is an important signaling pathway that regulates cell proliferation and apoptosis *via* murine double minute 2 (Mdm2)/tumor protein p53 (p53) and Caspase-3 signaling pathways. The upregulation of the G0/G1 phase and concurrent decrease in the S phase of breast cancer cells inhibited tumor cell proliferation and promoted apoptosis *via* AKT inactivation during the cell cycle (Tan et al., 2016). PPS inhibited the proliferation of lung cancer A549 cells by altering the intracellular localization of human antigen R (HuR) protein (*i.e.*, decreased cytoplasmic HuR protein expression and increased nuclei HuR protein expression), decreasing Cyclin D1 mRNA stability, and reducing Cyclin D1 protein expression. MTT and FCM assays also revealed that PPS significantly limits the proliferation of A549 tumor cells (Shen, Xu, & Zeng, 2016). HPP enhanced the secretion of inflammatory factors TNF-*α*, IL-1*β*, and iNOS in the macrophage model (THP-1 human leukemic cells induced by 100 ng/mL phorbol myristate acetate) and upregulated the expression of surface molecules CD16, CD23, CD40, and CD86 in macrophages, polarizing them toward the M1 phenotype. The HPP-polarized macrophage-derived conditioned medium was employed to investigate the impact of macrophages on human bladder cancer cells T24 and EJ. Activated macrophages suppressed bladder cancer cell proliferation, regulated apoptosis, and inhibited migration and epithelial-mesenchymal transition (EMT) by downregulating the Janus kinase 2 (JAK2)/NF-κB pathway. Consequently, this inhibition decreased bladder cancer progression (Jia et al., 2021).

The antitumor activity of PUPs may also be attributed to additional mechanisms. *In vitro* studies have demonstrated that PUP treatment effectively downregulated immunosuppression in tumor cells, as evidenced by a significant reduction in transforming growth factor beta 1 (TGF-*β*1) levels in rectal cancer-derived Colon26 cells. PUP mitigated tumor cell-mediated immunosuppression by suppressing the secretion of the immunosuppressive molecule TGF-*β*1, thereby exerting its antitumor effect (Cui et al., 2009).

**Fig. 5:**
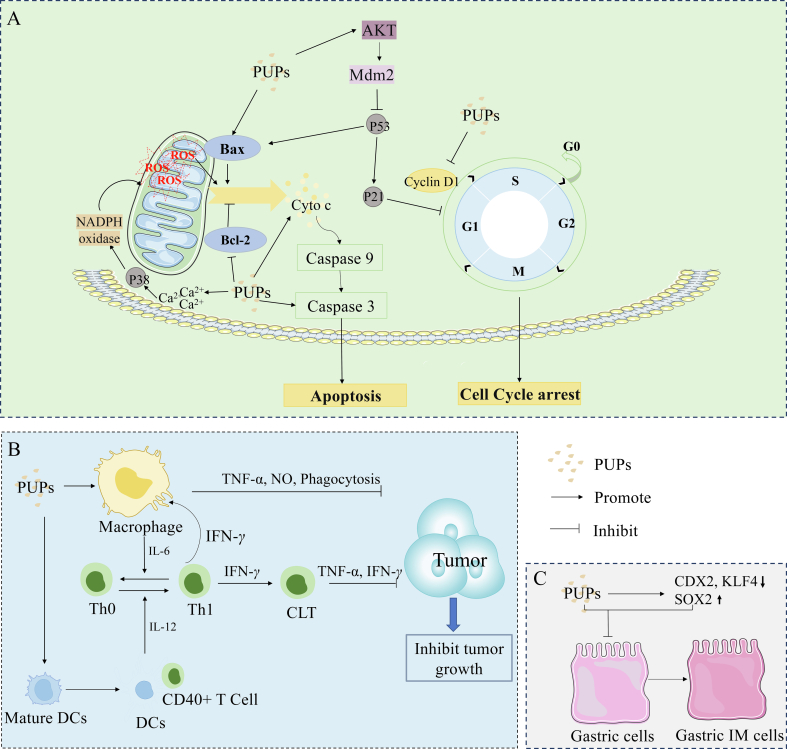
Mechanism of PUPs in combating tumors. A: PUPs exert a direct impact on tumor cells, inducing apoptosis and inhibiting tumor formation and progression. The direct anti-tumor effect of PUPs involves arresting the cell cycle and triggering apoptosis. B: Diverse immune cells interact to establish an immune regulatory network that effectively hinders tumor growth and metastasis. C: PUPs downregulate the expression levels of CDX2 and KLF4 while maintaining SOX2 expression, thereby reversing the abnormal transformation of normal gastric mucosal epithelial cells.

PPS treatment inhibited caudal type homeobox 2 (CDX2) and *Homo sapiens* Kruppel-like factor 4 (KLF4) and maintained the expression of Sex determining region Y (SOX2) IM cell models (gastric mucosal epithelial cells were replaced by intestinal epithelial cells), and played a protective role in gastric mucosal epithelial cells. This application reversed the effect of chenodeoxycholic acid (CDCA) on the IM transformation of normal gastric mucosal epithelial cells after stimulation (Lin, Feng, Cui, & Wei, 2021). The anti-cancer effects of PPS were related to regulating the activity of phase II metabolizing enzymes (GST, NQ01, *etc.*). PPS treatment decreased cytochrome P450 (CYP450) activity and increased the activity of NADP(H) quinone REDOX reductase NQ0l in a BBN and Saccharin-induced bladder cancer rat model, playing an antitumor role (Zhang et al., 2011). The anti-tumor mechanisms of PUPs are displayed in Fig. 5.

### 5.3 Antioxidant activity

The antioxidant activities of PUPs have been extensively documented, encompassing (1) the enhancement of scavenging ability against 2,2-azinobis-3-ethylbenzothiazoline-6-sulfonic acid (ABTS) and 2,2-diphenyl-1-picrylhydrazyl (DPPH) free radicals, (2) the augmentation of iron-reducing antioxidant capacity, and (3) the elevation in scavenging ability toward superoxide anions and hydroxyl radicals.

Three PUPs isolated from fermentation liquid, PPS1 (6.90 × 10^4^ Da), PPS2 (4.60 × 10^4^ Da), and PPS3 (3.70 × 10^4^ Da), showed different antioxidant activities. The scavenging ability of the three polysaccharides to DPPH was similar, with half-maximal inhibitory concentration (IC_50_) values of 4.0, 2.5, and 1.2 mg/mL, respectively. Their clearance rates of ABTS^+^ (2.0 mg/mL) were 19.5, 39.5, and 75.63 %, with IC_50_ values of 5.5, 2.5, and 1.5 mg/mL, respectively (Liu et al., 2019).

The scavenging effect of GUMP-1-2 on hydroxyl and superoxide radicals and the chelating effect on ferrous ions exhibited a dose-dependent relationship. The superoxide radical scavenging ability of GUMP-1-2 was significantly enhanced within the concentration range of 0 to 8 mg/mL. Particularly, the superoxide radical scavenging capacity of GUMP-1-2 approached that of ascorbic acid at a dosage of 8 mg/mL. Furthermore, the chelation capacity reached its peak at a concentration of 8 mg/mL with a remarkable chelation rate of 96.5 %. Conversely, neutral polysaccharide GPUM-1-1 demonstrated comparatively weaker *in vitro* antioxidant activity across all concentrations than acidic polysaccharide GPUM-1-2 (Bi et al., 2013). The scavenging activities of PUP60S2 against hydroxyl, DPPH, and superoxide radicals (2.00 mg/mL) were 62.36, 76.13, and 74.32 %, with IC_50_ values of 0.85, 0.53, and 0.74 mg/mL, respectively (He et al., 2015). The antioxidant activities of PUP60S2 and PUP80S1 were compared using the DPPH method and the oxygen radical absorbance capacity (ORAC) assay. Both PUP60S2 and PUP80S1 exhibited good antioxidant capacity, with ORAC values of 958.32 ± 26.05 and 812.65 ± 25.12 μmol TE/g, respectively, which were higher than those of other reported polysaccharides. The DPPH assay revealed that the maximal clearance of PUP60S2 and PUP80S1 at 2.0 mg/mL was 76.13 ± 2.97 and 57.21 ± 3.07 %, with IC_50_ values of 0.53 and 0.93 mg/mL, respectively, which were both dose-dependent. PPS exhibited a protective effect against carbon tetrachloride (CCl4)-induced liver injury, potentially through the upregulation of superoxide dismutase (SOD), catalase (CAT), and glutathione peroxidase (GPx) levels (Kang et al., 2012). PB effectively eliminated free radicals and hindered lipid peroxidation *in vivo* by enhancing the total antioxidant capacity (T-AOC) and glutathione peroxidase (GSH-Px), elevating SOD and glutathione (GSH) levels, and reducing malondialdehyde (MDA) content (Du et al., 2014). The antioxidative properties of PUPs may be associated with their ability to protect against radiation damage. Administration of PUPs at a dosage of 50 mg/kg significantly reduced comet tail length in irradiated mice peripheral blood leukocytes, thereby mitigating oxidative DNA damage (8-hydroxy-2-deoxyguanosine) and inhibiting lipid peroxidation formation (Wu et al., 2011).

### 5.4 Other biological activities

PPS inhibited myofibroblast differentiation through the TGF-*β*1-induced Smad2/3 signaling pathway and significantly reversed the increase in alpha-smooth muscle actin (*α*-SMA) and collagen production after TGF-*β*1 stimulation of human lung fibroblasts (HLFs) (Jiang et al., 2020). PPUS therapy effectively mitigated renal fibrosis in a unilateral ureteral obstruction (UUO) renal fibrosis model by suppressing inflammation and restoring the equilibrium between matrix metalloproteinases (MMPs) and tissue inhibitors of metalloproteinases (TIMPs), thereby impeding EMT. The restoration of MMPs/TIMPs balance re-establishes the predominance of antifibrotic factors over fibrotic factors, ultimately diminishing extracellular matrix production and deposition in renal tissue, thus ameliorating collagen deposition and reducing the extent of fibrosis (Li et al., 2019).

A study of the regulatory effects of PPS on lipopolysaccharide (LPS)-induced cytokines in the J774 macrophage inflammation model revealed that PPS effectively attenuated the upregulation of LPS-induced inflammatory factors and suppressed the phosphorylation of mitogen-activated protein kinases (MAPKs) p38 and p65 and extracellular signal-regulated protein kinases 42/44 (ERK42/44). Moreover, PPS mitigated inflammatory damage by modulating the MAPK signaling pathway (Jiang, Li, Zhao, & Zeng, 2015). PPUS ameliorated systemic inflammation during renal fibrosis by reducing inflammatory cell infiltration in rat kidney tissue and downregulating TNF-*α* and IL-1*β* expression. Additionally, PPUS alleviated inflammation by inhibiting EMT, ultimately improving renal collagen deposition and reducing fibrotic severity (Li et al., 2019).

The hypoglycemic activity of PPS was demonstrated through a streptozotocin (STZ)-induced diabetic rat model. Compared with the diabetic control group, PPS markedly enhanced the activities of hepatic glucokinase (GCK), pyruvate kinase (PK), and hexokinase (HK) and the expression of glucose transporter type 4 (GLUT4). Moreover, PPS reduced the activity of glucose-6-phosphatase (G-6-Pase) and the expression of phosphoenolpyruvate carboxykinase (PEPCK) in the liver. PPS can reduce the progression of gluconeogenesis and increase hepatic glycolysis, thereby promoting glucose metabolism in diabetic rats (Zhang, Luo, & Luo, 2020).

PPS (100, 200, 400 mg/kg) reduced the rate of cyclophosphamide-induced micronuclei in mouse bone marrow cells, with an 84.28 % reduction at a dose of 100 mg/kg, demonstrating a negative correlation between dosage and effect. Furthermore, PPS exhibited a dose-dependent decrease in the rate of sperm malformation in mice, with a 60.40 % reduction at a dose of 400 mg/kg, indicating a positive correlation with dosage. These findings highlight the anti-mutagenic properties of PPS and its ability to safeguard genetic material integrity while providing protective effects on mammalian germ cells (Wang, Liu, & Shao, 2014).

PUP injection was employed as a course of therapy for 156 patients with hepatitis B for 3 months, demonstrating a significant effect. The negative conversion rate of hepatitis B e-antigen (HBeAg) in patients with different hepatitis B subtypes after one course of treatment was 44.68 % in patients with chronic active hepatitis (CAH), 34.85 % in those with chronic persistent hepatitis (CPH), and 28.57 % in those with post-hepatitis cirrhosis (LC) (Liu, Yuan, Wang, Yang, & Zhou, 1993). Oral PUP capsules 3 times a day, 2 capsules each time, for 3 months as a course of treatment for 28 patients with chronic hepatitis B demonstrated significant efficacy. The negative rate of HBeAg, hepatitis B surface antigen (HBsAg), and HBV-DNA was 64.7, 10.7, and 63.2 %, respectively. The positive rate of anti-HBeAb was 52.9 % (Chen & Luo, 2001).

The mycelial polysaccharide extract PUm-C from *P. umbellatus* demonstrated inhibitory effects on the proliferation of *Escherichia coli* (*E. coli*) and *Staphylococcus aureus* (*S. aureus*), exhibiting an increasing size of the inhibition zone with higher dilutions of the seed bacteria. However, no inhibitory activity was observed against *Aspergillus niger* and *Aspergillus nidulans* (Sun & Zhou, 2014).

RAW264.7 cells were stained with senescence-associated *β*-galactosidase (SA-*β*-Gal) after 48 h of treatment with D-Gal (200 mg/mL). The D-Gal group exhibited more positive blue-stained cells than the control group; however, cellular aging indices based on *β*-galactosidase activity in the PPS1, PPS2, and PPS3 groups were all significantly lower than those in the D-Gal group (*p < 0.01*). Therefore, it was inferred that PPS1, PPS2, and PPS3 reduced the level of the senescence marker *β*-galactosidase in RAW264.7 cells, thereby delaying cellular senescence. PPS1, PPS2, and PPS3 also protect against hydrogen peroxide (H_2_O_2_) + ultraviolet (UV)-induced DNA damage. Supercoiled (SC) DNA undergoes cleavage in the presence of H2O2 and UV irradiation, generating open circular (OC) DNA and linear (L) DNA, ultimately decreasing the electrophoretic density of SC DNA. DNA electrophoretic density analyses showed that treatment with PPS1, PPS2, and PPS3 significantly reversed the H_2_O_2_ + UV-induced decrease in SC DNA density (*p < 0.01*) (Liu et al., 2019).

## 6 Structural-activity relationships of PUPs

The biological activities of polysaccharides are closely influenced by their chemical structure characteristics, such as molecular weight, composition of monosaccharides, uronic acid content, type of glycosidic bond, branched chain structure, and structural modification and conformation. However, studies on the correlation between the structures of PUPs and their biological activities are scarce due to their intricate nature. Therefore, this section explores in depth the intricate relationship between the structures of PUPs and their biological activities based on existing studies that have (Table 5).

### 6.1 Molecular weight (Mw) effects

PPS1 (6.90 × 10^4^ Da), PPS2 (4.60 × 10^4^ Da), and PPS3 (3.70 × 10^4^ Da) were obtained from *P. umbellatus* fermentation liquid. The low molecular weight PPS3 (3.70 × 10^4^ Da) showed better antioxidant activity. DPPH scavenging capacities of the three polysaccharides were as follows: PPS3 (IC_50_: 1.2 mg/mL) > PPS2 (IC_50_: 2.5 mg/mL) > PPS1 (IC_50_: 4.0 mg/mL). The clearance rates of ABTS^+^ (2.0 mg/mL) were 75.63 % (IC_50_: 1.5 mg/mL) for PPS3, 39.5 % (IC_50_: 2.5 mg/mL) for PPS2, and 19.5 % (IC_50_: 5.5 mg/mL) for PPS1 (Liu et al., 2019). These results indicate that the low molecular weight PPS3 has high antioxidant activity, consistent with the reports of Cheung, Siu, Liu, and Wu (2012) and Wang, Hu, Nie, Yu, and Xie (2016) that lower molecular weight of polysaccharides correlates with stronger antioxidant activity.

### 6.2 Uronic acid and branching structure effects

Bi et al. (2013) assessed the antioxidant potential of GUMP-1-1 and GUMP-1-2 through *in vitro* experiments, including hydroxyl radical scavenging, superoxide radical scavenging, and chelating effect on ferrous ions. The findings indicated that the neutral polysaccharide GUMP-1-1 exhibited lower activity than its acidic counterpart GUMP-1-2 across all tested concentrations. Moreover, PUPs with a high degree of branching and uronic acid content also showed better antioxidant activity. He et al. (2016) compared the antioxidant activity of PUP60S2 and PUP80S1 using the DPPH method and the ORAC assay and found that PU60S2 with higher branching and uronic acid content exhibited stronger antioxidant activity. The ORAC value was 958.32 ± 26.05 μmol TE/g for PUP60S2 and 812.65 ± 25.12 μmol TE/g for PUP80S1. The DPPH assay results showed that the maximum clearance rate was 76.12.97 % (IC_50_: 0.53 mg/mL) for PUP60S2 and 57.21 ± 3.07 % (IC_50_: 0.93 mg/mL) for PUP80S1. PUP60S2 was also superior to PUP80S1 in the scavenging experiments of superoxide and hydroxyl free radicals. Collectively, these results suggest that the antioxidant activity of PUPs is related to their uronic acid, and PUPs containing uronic acid have better antioxidant capacity.

### 6.3 Glycosyl linkage type and branching structure effects

Studies exploring the interplay between the structural configurations of fungal polysaccharides and their immunomodulatory and antitumor activities indicate that polysaccharides featuring a predominately 1,3-*β*-D-Glc*p* main chain exhibit pronounced antitumor effects (Ferrira, Vaz, Vasconcelos, & Martins, 2010; Wasser, 2002).

Importantly, the antitumor efficacy is not only influenced by the structure of the main chains but also by the positioning and the extent of branching. GU-2, GU-3, and GU-4 polysaccharides were administered *via* intraperitoneal injection (1 or 5 mg/kg/day, for 10 days) in mouse S-180 sarcoma cells (subcutaneously implanted, 30 mg). GU-3, containing 1,3-*β*-D-Glc*p* and branching at C-6, exhibited strong antitumor activity (5 mg/kg/day, tumor regression at 42 days, 86.2 %). In addition, the antitumor activity was not only related to the backbone structure but also to the solubility of the polysaccharide and the length of the branch chain (Miyazaki et al., 1979). Ueno, Abe, Yamauchi and Kato, 1980, Ueno, Okamoto, Yamauchi and Kato, 1982 studied the antitumor activity of the water-soluble polysaccharide WP and the alkali-soluble polysaccharide AP and its derivatives with backbone structure of 1,3-*β*-D-Glc*p* in mouse S-180 sarcoma cells. It was found that the inhibition rate was 100 % for WP and 57–100 % for AP and its derivatives in the 5th week. Li et al. (1995) evaluated the inhibitory activity of the water-soluble polysaccharide P1 and the alkali-soluble polysaccharide P2 (with a backbone structure of 1,3-*β*-D-Glc*p*) and reported that the tumor inhibitory rate was 80.2 % for P1 and 16.1 % for P2. The tumor inhibition rate was 87.4 % for P1 and 24.2 % for P2 after removing the side chain. The unique *β*-glucan backbone structure of ZPS ensures contact with B-cell antigen receptors and/or pattern recognition receptors, with a higher degree of side-chain removal significantly enhancing its capacity to reduce B-cell proliferative activity *in vitro* (Dai et al., 2012).

### 6.4 Functional structure effects

Polysaccharides possess numerous derivable groups such as hydroxyl, ketone, and aldehyde groups along their molecular chains. Polysaccharides acquire specific structures and properties by adding different chemical groups, and various polysaccharide derivatives are produced by chemical modification. Notably, although chemical modification can enhance the biological functions of PUPs, the effectiveness of such modifications may vary depending on the specific method employed (Liu, Jiao, Wang, Zhou, & Zhang, 2008; Xie et al., 2019). Selenium polysaccharide is an organic selenium compound combined with selenium and polysaccharide. Selenium polysaccharide has a special chemical structure, the seleno‑oxygen bond (O=Se = O), which is different from an ordinary polysaccharide. It demonstrates a synergistic effect by combining the properties of selenium and polysaccharide, resulting in significantly enhanced activity compared with individual selenium or polysaccharide (Jin, Yan, Nian, & Tang, 2021). PUP-SeNPs were prepared from PUPs and sodium selenite by chemical synthesis (Li, Liu, Wang, Deng, & Li, 2013). The inhibitory effect of PUP-SeNPs was stronger than that of PUPs on *E. coli*, *S. aureus*, brewer's yeast, and *Aspergillus niger*. Zinc-modified PUPs showed stronger antioxidant capacity *in vitro* than unmodified PUPs. PUPs and PUP‑zinc exhibited a good scavenging effect on hydroxyl radical and superoxide anion radical in *in vitro* antioxidant experiments, and the scavenging ability was positively correlated with the added dose. The clearance rate of ·O^2−^ produced by the AP-TEMED system was 66.26 % for PUP‑zinc and 64.3 % for PUPs. The clearance rate of ·OH^−^ produced by H_2_O_2_/Fe system was 65.2 % for PUP‑zinc and 63.04 % for PUPs (Li, 2011; Liu et al., 2008).

Liu et al. (2013) used a sulfonic acid-pyridine reagent for sulfated-PUPs, and then sulfated-PUPs were obtained by Sephadex G-75 and dialysis. Then, using the Fenton reaction (H_2_O_2_ and Fe^2+^ produce ·OH^−^), salicylic acid was added to the system to capture ·OH^−^, which produced colored substances with maximum absorption at 510 nm. Sulfated-PUPs showed a higher scavenging effect on ·OH^−^ than unmodified PUPs. Sulfation of polysaccharides has become a crucial direction of structural modification of polysaccharides, as sulfated polysaccharides exhibited new biological activities (Xie et al., 2019). Furthermore, PUPs produced certain anticoagulant activity after sulfated modification. The fresh rabbit blood method was applied to detect the degree of coagulation. It was found that sulfated-PUPs had certain anticoagulant activity compared with the heparin standard group, and the titer was calculated to be 45.47 U/mg, which was 30.31 % of the heparin standard (Li, 2012).

## 7 Applications

*P. umbellatus* sclerotia are frequently utilized in Traditional Chinese Medicine (TCM) alone or in combination with other herbs to effectively eliminate dampness and diuresis and reduce swelling. In 2018, they were included in the prestigious directory of certified organic foods featured on the official website of the Certification and Accreditation Administration of the People's Republic of China (CNCA) (https://www.samr.gov.cn). Moreover, various TCM formulations containing *P. umbellatus* sclerotia or its extract have been incorporated into the 2020 edition of the Chinese Pharmacopoeia, including Zhuling tang, Fenxiao tang, Wuling powder, Yinchen Wuling powder, Zhuling wan, *etc.* (Commission, 2020). As the core active ingredient, PUPs play a key role in the pharmacodynamic activity of *P. umbellatus*. The inhibitory effect of PUPs on tumors was discovered in 1975 (code-named 757) (Zheng, 1980). Since 1990, PUPs have been commercially produced as an immunomodulator for the treatment of hepatitis in China (Zong, Cao, & Wang, 2012) and almost no obvious toxicity and side effects have been reported. Currently, the commonly used medicinal products of *P. umbellatus* polysaccharide include PUP capsules and PUP injection (with PUPs as the only raw material), which are approved by the National Medical Products Administration (NMPA) (https://www.nmpa.gov.cn). These products are utilized for the treatment of chronic hepatitis B, anti-tumor therapy, and immune regulation.

Additionally, PUPs possess safe, low irritant properties, and exhibit antioxidant, anti-UV radiation, and antibacterial activities, which are the basic properties required for most cosmetics, indicating that PUPs have good application potential in the cosmetics industry. PPS1, PPS2, and PPS3 polysaccharides extracted from the *P. umbellatus* sclerotia fermentation liquid can reduce the level of *β*-galactosidase senescence markers, delay cell senescence, and protect the DNA of human skin cells from UV radiation, underscoring their role in anti-aging and preventing skin photoaging (Liu et al., 2019), and thus can be potentially used in anti-aging and prevention of melasma formation. PUm—C, extracted from the mycelium of *P. umbellatus*, inhibited the growth of *E. coli* and *S. aureus* (Sun & Zhou, 2014). The findings indicate that PUm-C may serve as a substitute preservative in cosmetic products, effectively impeding the proliferation of microorganisms. Additionally, it can be utilized as an active component against acne by inhibiting the growth of associated bacteria.

Accumulating evidence has demonstrated that PUPs are the core active substance of *P. umbellatus*. With the continuous progress of PUP research, PUP products are more likely to become available and show greater prospects for use in functional foods, dietary supplements, medicines, and cosmetics. It is noteworthy that as products for clinical use, PUPs efficacy and safety have been proven. Therefore, developing PUPs-related products and promoting the accurate utilization of PUPs is imperative.

## 8 Concluding remarks and prospects

*P. umbellatus*, a valuable medicinal fungus known to treat various diseases, has been extensively investigated in recent years with regard to its extraction, isolation, purification, structural identification, and biological activity its component (PUPs). Although current research on PUPs has yielded important findings, several aspects remain to be clarified. The pharmacological activity and structural characteristics of polysaccharides are highly dependent on the stable preparation process. Different raw materials, extraction, separation, and purification methods may affect the structure, spatial conformation, and biological activity of PUPs. Nowadays, the commonly used extraction methods for PUPs include traditional water extraction, ultrasonic or microwave- assisted extraction, with water extraction being the most widely used. However, the traditional water extraction method cannot meet the technical, quality stability, and pharmacological activity requirements of PUPs. Therefore, new extraction methods, such as enzyme extraction or even a combination of multiple extraction methods, should be adopted to establish environmentally friendly, efficient, and stable standardized process to ensure green, high-yield and reproducible PUPs preparation.

Due to the complex structure and technological limitations, quality control of PUPs remains to be a challenge. Considering that PUPs is an important active component of *P. umbellatus*, researchers should develop a suitable method to facilitate the qualitative and quantitative analysis of PUPs in *P. umbellatus-*related products. For instance, based on the chemical properties of PUPs presented in Table 2, Table 3, Table 4, its molecular weight distribution (6.88–2270 kDa), monosaccharide composition (including Glc, Man, Gal, Fru and GlcA), and glycosidic bond (including 1,3-*β*-D-Glc*p*, 1,4-*β*-D-Glc*p*, 1,6-*β*-D-Glc*p*) are used as potential indices to evaluate the quality of PUPs. Moreover, the molecular weight distribution and types of monosaccharide composition as well as their proportions in PUPs can be analysed using alternative techniques such as HPGPC-RID and HPLC-ELSD. Furthermore, the routine analysis of glycosidic linkages can be efficiently achieved by integrating saccharide mapping with bioassay techniques.

**Table 3:** Constituent monosaccharides of PUPs and chromatographic methods used for their determination.

PUPs	Molar ratios of constituent monosaccharides	Related Methods				Refs.
		Hydrolysis	Derivative	Column	Detection	
**Gas chromatography**						
ZPS	Glc (>90 %)	2 M TFA, 100 °C, 2 h	Ac_2_O	Gas-Chrom P column	FID	(Dai et al., 2012)
GUMP-1-1	Glc: Man: Gal: Fru = 17.4: 1.4: 1.2: 0.4	2 M TFA, 120 °C, 3 h	Ac_2_O	HP-5 column	FID	(Bi et al., 2013)
GUMP-1-2	Glc: Man: Gal: Fru: GlcA =11.4: 1.2: 1.1: 0.2: 2.1					
PUP60S2	Glc 77.7 %, GlcA 22.3 %	2 M TFA, 110 °C, 2 h	Ac_2_O	HP-5 capillary column	FID	(He et al., 2015)
PUP80S1	Glc 91.5%, GlcA 8.5%	2 M TFA, 110 °C, 2 h	Ac_2_O	HP–5 capillary column	FID	(He et al., 2016)
PUP60W-1	Fuc: Glc: Gal = 1.0: 0.9: 13.3	2 M TFA, 110 °C, 2 h	Ac_2_O	HP–5 capillary column	FID	(He et al., 2017)
PPUS	Gal: Fuc: Glc = 13.24: 1.03: 0.88	2 M TFA, 110 °C, 2 h	Ac_2_O	HP–5 capillary column	FID	(Li et al., 2019)
HPP	Glc	4 M TFA, 120 °C, 4 h	PMP	DB-1701 silica capillary column	MS	(Liu et al., 2020)
**High-performance liquid chromatography**						
PUm-C	Glc: Gal = 1.57: 1	TFA	Not Detected	Not Detected	UV	(Sun & Zhou, 2014)
PUf-C	Glc: Gal = 5.42: 1					
PPS	Glc 62.28 %，Trehalose 6.05 %	Not Detected	Not Detected	Not Detected	Not Detected	(Zhang et al., 2015)
PPS1	Man: Gal: Glc = 43.6: 2.5: 1.0	2 M TFA, 122 °C, 100 min	PMP	WondaSil C18 column	UV	(Liu et al., 2019)
PPS2	Man: Gal: Glc = 17.7: 3.1: 1.0					
PPS3	Man:Gal:Glc = 4.6: 2.6: 1.0					
HPP	Glc	4 M TFA, 120 °C, 4 h	PMP	HypersiL BDS C_18_ column	UV	(Jia et al., 2021; Liu et al., 2020)
**Paper chromatography**						
GU-0	Glc、Gal、Man、Xyl、GlcA	1 N H_2_SO_4_, 100 °C, 5 h	Ac_2_O	Not Detected	Filter Paper	(Miyazaki & Oikawa, 1973)
GU-1	Glc、GlcA 2.6%	1 N H_2_SO_4_, 100 °C, 6 h	Ac_2_O			
WP	D-Glc、D-Man、D-GlcA	0.5 M sulfuric acid overnight, 100 °C	Not Detected			(Ueno et al., 1982)
ZP	D-Glc、D-Man					
AP-1 ∼ AP-10	Glc					
**Gas-liquid chromatography (GLC)**						
AP	D-Glc	1 M HCl in methanol, 100 °C, 24 h	TMS-HT	3 % ECNSS-M Glass column	FID	(Ueno et al., 1980)

**Table 4:** Glycosidic linkages of PUPs, methylation and GC–MS used for their determination.

PUPs	Backbone	Substituted sugar unit	Molar ratio	Related Methods		Refs.
				Methylation conditions	Analytical method	
GU-1	1 → 3-*β*-Glc*p,* 1 → 4-*β*-Glc*p*, 1 → 6-*β*-Glc*p*	Terminal-Glc*p*; 1,6-linked-Glc*p*; 1,4-linked-Glc*p*; 1,3-linked-Glc*p*	1.23: 2.63: 1.00	DMSO-NaH-CH_3_I; 0.5 N CH_3_OH-HCl, 100 °C, 10 h; HCOOH (90 %), 100 °C, 10 h; 1 N H_2_SO_4_ 100 °C, 4 h	GLC (BDS column) TLC (Paper electrophoresis)	(Miyazaki & Oikawa, 1973)
AP	1 → 3-*β*-Glc*p*	Terminal-Glc*p*; 3-linked-Glc*p*; 3,6-linked-Glc*p*	1.0: 2.0: 1.0	DMSO-NaH-CH3I; HCOOH (90 %), 100 °C, 2 h; 0.25 M H_2_SO_4_ 100 °C, 12 h	GLC (3 % ECNSS-M glass column) GLC-MS (2 % OV-1glass column)	(Ueno et al., 1980)
ZPS	1 → 6-*β*-Glc*p*, 1 → 4-*β*-Glc*p*	1,6-linked-Glc*p*; 1,3,6-linked-Glc*p*; 1,4-linked-Glc*p*; 1,3-linked-Glc*p*; Terminal-Glc*p*	9.25: 8.29: 9.35:7.90: 8.65	DMSO-NaOH-CH_3_I; HCOOH (88 %), 100 °C, 3 h; 2 M TFA, 110 °C, 6 h；Ac_2_OH	GC–MS (DB-5 column)	(Dai et al., 2012)
PUP60S2	1 → 6-*β*-Glc*p*	Terminal-Glc*p*; 3-linked-Glc*p*; 4-linked-Glc*p*; 6-linked-Glc*p*; 3,6-linked-Glc*p*	0.50: 0.60: 0.25: 1.00: 0.78	DMSO-NaOH-CH_3_I; HCOOH (88 %), 100 °C, 3 h; 2 M TFA, 110 °C, 6 h；Ac_2_OH	GC–MS (HP-5 capillary column)	(He et al., 2015)
PUP80S1	1 → 3-*β*-Glc*p*, 1 → 6-*β*-Glc*p*	Terminal-Glc*p*; 1,3-linked-Glc*p*; 1,4-linked-Glc*p*; 1,6-linked-Glc*p*; 1,3,6-linked-Glc*p*	1.16: 1.63: 0.82: 4.11: 1.00	DMSO-NaOH-CH_3_I; HCOOH (88 %), 100 °C, 3 h; 2 M TFA, 110 °C, 6 h; Ac_2_OH	GC–MS (HP–5 capillary column)	(He et al., 2016)
PUP60W-1 PPUS	1 → 6-*α*-Glc*p*	1,6-linked-Fuc*p*; 1-linked-Gal*p*; 1,3-linked-Glc*p*; 1,4-linked-Glc*p*; 1,2-linked-Gal*p*; 1,6-linked-Glc*p*; 1,6-linked-Gal*p*; 1,2,3-linked-Gal*p*; 1,2,6-linked-Gal*p*	0.62: 4.12: 0.28: 0.32: 0.45: 0.38: 2.03: 1.00: 4.45	DMSO-NaOH-CH_3_I; HCOOH (88 %), 100 °C, 3 h; 2 M TFA, 110 °C, 6 h; Ac_2_OH	GC–MS (HP–5 capillary column)	(He et al., 2017; Li et al., 2019)
PUP-W-1	1 → 3-*β*-Glc*p,* 1 → 6-*β*-Glc*p*	Terminal-Glcp; 1,3-linked-Glc*p*; 1,6-linked-Glc*p;* 1,3,6-linked-Glc*p*	Molar percentages of 8.29 %, 60.11 %, 25.18 %, and 6.42 %	DMSO-NaOH-CH_3_I; HCOOH (88 %), 100 °C, 3 h; 2 M TFA, 110 °C, 6 h; Ac_2_OH	GC–MS (Trace TR-5MS capillary column)	(Gao et al., 2023)

**Table 5:** Chemical structure characteristics and the biological activity of PUPs.

Fraction	Structure	Model	Function	Mechanism	Ref.
GU-2	Backbone composed of (1 → 6)-*β*-Glc*p* and (1 → 3)-*β*-Glc*p*, branches composed of (1 → 6)-*β*-Glc*p* and (1 → 4)-*α*-Glc*p*.	Sarcoma 180 cells ICR-JCL mice (Female, 5 week) (*in vivo*)	Antitumor	Inhibit the growth of sarcoma 180 in mice	(Miyazaki et al., 1979)
GU-3	Backbone composed of (1 → 3)-*β*-Glc*p* and (1 → 6)-*β*-Glc*p* and branching at C-6 position				
GU-4	Backbone composed of (1 → 3)-*β*-D-Glc*p*, branches at C-6 position				
AP-3		Sarcoma 180 cells STD-ddY Mice (Female, 5 week) (*in vivo*)			(Ueno et al., 1982)
PPS	Not Detected	H_2_O_2_/Fe^2+^ system method (*in vitro*)	Antioxidant	Scavenging ·OH^−^	(Liu et al., 2013)
PPS	Absorption peak of a *β*-D-Glc*p*	Bone marrow cells, Splenocytes (*in vitro*)	Immunomodulate	Increase IL-12 production by BMDCs, activation and maturation of murine BMDCs Increase spleen index and splenocyte proliferation	(Li et al., 2010)
PPS	Absorption peak of a *β*-D-Glc*p*	Spleens, Peritoneal macrophages (*in vitro*)	Immunomodulate	Increase splenocyte proliferation Increase TNF-*α*、IL-1*β* and NO levels	(Li & Wen, 2011)
HPP	Not Detected	Use Phorbol myristate acetate (PMA) to induce THP-1 human leukemic cells as a macrophage model	Immunomodulate	Increasing IL-1*β*, TNF-*α* and iNOS, CD86, CD16, CD23, and CD40 level, polarizing macrophages to M1 type Downregulating JAK2/NF-*κ*B pathway	(Jia et al., 2021)
PPS	Not Detected	BBN-induced Fisher-344 bladder cancer rats (female) (*in vivo, in vitro*)	Immunomodulate	Protection against mouse spleen and thymus index Increasing peritoneal macrophages D86, D40 and TLR4/D14 levels	(Zhang et al., 2015)
PB	Not Detected	Breast cancer cells BALB/c nu/nu nude Mice (male, 4- 6 week) (*in vivo*)	Antitumor	Decreasing AKT, increasing Caspase-3	(Tan et al., 2016)
PPS	Not Detected	HLFs cell (*in vitro*) BLM-induced pulmonary fibrosis C57BL/6 mice Model (Male, 6–8 weeks) (*in vivo*)	Anti-fibrosis	Inhibiting myofibroblast differentiation through TGF-*β*1-induced Smad 2/3 pathway	(Jiang et al., 2020)
ZPS	DB 44.5%	Splenocytes, splenic T, B and peritoneal macrophages (*in vitro*)	Immunomodulate	Activating macrophages cells, increase IL-1*β* levels Increase splenocyte and spleen B cells proliferation	(Dai et al., 2012)
GUMP-1-1	Not Detected	Hydroxyl radical activity, superoxide radical activity, metal chelating assay and spleen cells (*in vitro*) BALB/c mice inoculated with H22 tumor cells (half male and half female) (*in vivo*)	Antioxidant Antitumor Immunomodulate	Scavenging ·OH^−^, ·O^2−^, iron reduction, *etc.* Reducing the tumor volume of H22 tumor-bearing mice Increasing spleen index and splenocyte proliferation	(Bi et al., 2013)
GUMP-1-2					
PUm-C	Not Detected	Spleen lymph cells and *escherichia coli*, *staphylococcus aureus*, aspergillus niger, aspergillus nidulans (*in vitro*)	Immunomodulate Antibacterial	Increasing NK and LAK cell lethality, promoting B cell and T cell proliferation Inhibiting the proliferation of *escherichia coli* and *staphylococcus aureus*	(Sun & Zhou, 2014)
PUf-C		Spleen lymph cells (*in vitro*)	Immunomodulate	Increasing NK and LAK cell lethality, promoting B cell and T cell proliferation	
PUP60S2	DB 40 %	DPPH radical activity, hydroxyl radical activity, superoxide radical activity (*in vitro*)	Antioxidant	Increasing ORAC, scavenging ·OH^−^, DPPH, ·O^2−^	(He et al., 2015)
PUP80S1	DB around 22.8%	ORAC assay, DPPH radical activity (*in vitro*)	Antioxidant	Increasing ORAC, Scavenging ·OH^−^, DPPH, ·O^2−^	(He et al., 2016)
PPUS		UUO induced-Balb/c renal fibrosis mice model (Male, 6 week) (*in vivo*)	Anti-inflammatory	Decreasing IL-1*β*、TNF-*α*	(Li et al., 2019)
PPS	Not Detected	AP-TEMED system and H_2_O_2_/Fe^2+^ System method (*in vitro*)	Antioxidant	Scavenging ·OH^−^, ·O^2−^	(Li, 2011)
PPS1	Not Detected	Hydroxyl radical activity, DPPH radical activity, Superoxide radical activity, ABTS^+^ radical activity and RAW264.7 cell (*in vitro*)	Antioxidant Immunomodulate Retards cell senescence DNA damage protecting	Scavenging DPPH, ·OH^−^、·O^2−^, ABTS^+^, *etc.* Increasing the phagocytosis of macrophages and NO production Decreasing *β*-galactosidase aging marker in RAW264.7 Protecting the SC DNA from cleavage	(Liu et al., 2019)
PPS2	Residue A as →2,6) -α-Manp-(1→, B as →6)-Manp-(1→, C as →2)-Manp-(1→, D as →3)-Manp-(1→, E as Manp-(1→				
PPS3	Not Detected				
HPP		IFN-γ-stimulated RAW264.7 cells and treated RAW 264.7 cells with T24 cell culture supernatant to simulate the bladder tumor microenvironment (*in vitro*)	Immunomodulate	Increasing RANTES, IL-1*β*, IL-6, IL-23, NO level Increasing expression of M1 phenotype indicators CD80 *via* the NF-*κ*B and NLRP3 pathways	(Liu et al., 2020)

Nevertheless, the paucity of study on the conformational relationship and specific mechanism of action of PUPs severely limits their clinical applicability and development. The PUPs with lower molecular weight have stronger antioxidant properties (Liu et al., 2019), which can be explained by their enhanced intramolecular hydrogen bonding (O—H) and the presence of electron giving groups. Moreover, the bioactivity potential could be augmented by the heterogeneity of monosaccharide composition (He et al., 2016). Nonetheless, drawing definitive correlations between molecular weight, monosaccharide composition, and PUP activity is challenging, owing to the variability and inconsistencies in existing literature reports. The exact relationship between branch structure and function has not been fully explored so far. Therefore, it is imperative to perform extensive investigations to deeply understand the association between the branching configuration and efficacy of PUPs. This crucial step toward fully harnessing the therapeutic capabilities of these substances lies in acquiring and scrutinizing intricate structural data. Delving deeper into these areas, with a focus on elucidating conformational relationships, identifying active structural units, and clarifying specific mechanisms of action, would markedly advance the application and development of PUPs in future research initiatives.

In recent studies, PUPs have shown great potential to treat various diseases. However, most studies have focused on *in vitro* cells, enzymes, or chemical reactions, with little research on the pharmacokinetics of PUPs. This limits the utilization of PUPs in clinical practice. Several studies have elucidated the activities of PUPs, but most of them are based on pharmacodynamic evaluation and characterization of changes in some signaling pathways using *in vitro* cellular, enzymatic, or chemical assays. Owing to the limited repertoire of digestive enzymes encoded by human genes, polysaccharides, as macromolecular components, cannot be directly hydrolyzed and absorbed in the gastrointestinal enzymes following oral administration (Topping & Clifton, 2001). Such polysaccharides remain in the gut where they undergo degradation into small-molecule products by endogenous enzymes produced by the gut microbiota (Cabral et al., 2022). *β*-glucan is particularly abundant among the indigestible polysaccharides in PUPs and serves as one of its key representative polysaccharides. Their *β*-glucoside bonds are resistant to digestion by enzymes found in the human gastrointestinal tract but can be selectively metabolized by probiotic microorganisms like *Lactobacillus* and *Bifidobacterium* (Mironczuk-Chodakowska, Kujawowicz, & Witkowska, 2021; Niego et al., 2021). The low bioavailability of polysaccharides and the difficulty in achieving effective concentrations within target tissues undermine the credibility of pharmacodynamic results from many studies, particularly those based on *in vitro* experiments. Several researchers have documented the involvement of intestinal microorganisms and their metabolites in the regulation of various physiological functions of the organism, as well as the ability of some active molecules to regulate the physiological activities of intestinal microorganisms and their metabolites. These studies have laid a foundation for future investigations into the specific mechanism of action of PUPs. Furthermore, non-cellulosic *β*-glucans are recognized as biological response modifiers, exhibiting promising potential in immunomodulatory and antitumor applications. Given their diverse potential applications across various sectors, research into these compounds is expected to continue to expand. As a unique natural resource that has been extensively applied, and considering the increasingly sophisticated mycelium fermentation technology, *P. umbellatus* may become a sustainable source of *β*-(1 → 3)/(1 → 6)-glucan in future.

The health conditions in various parts of the contemporary society are decreasing, primarily attributed to the accelerated pace of life and poor dietary patterns (Das, Bhaumik, Raychaudhuri, & Chakraborty, 2012). Consequently, the demand for functional foods that improve the health status or prevent diseases is on the rise (Chandrasekara & Josheph, 2016; Chen, 2023). Due to the diverse range of activities and safety profile of *P. umbellatus*, its compounds are being utilized to develop functional foods, health supplements, and pharmaceuticals (Lu et al., 2020). Polysaccharides, classified as prebiotics, are selectively metabolized by host microorganisms to promote health (Das et al., 2012; Niego et al., 2021). Market acceptance and awareness can pose challenges for each polysaccharide product, particularly if the product is relatively unknown or raises potential safety concerns. However, the proven efficacy and safety of PUPs provide them with a competitive advantage over other fungal polysaccharides, where safety issues may arise. The use of PUPs is anticipated to experience significant growth in the coming years.

In summary, considering their proven efficacy and safety, sustainable sources, and abundant *β*-glucan-rich ingredients, PUPs may serve as functional foods, dietary supplements, as well as substrates for the pharmaceutical and cosmetic industries. This review comprehensively examines recent advancements in PUPs research, encompassing their preparation methods, chemical structure elucidation, biological activities, underlying molecular mechanisms, and structure-activity relationships. Furthermore, the current applications of PUPs are discussed. Finally, the review highlights promising avenues for future research, aiming to provide a valuable foundation for further investigations into the properties and applications of these bioactive compounds.

## CRediT authorship contribution statement

**Wei Gao:** Writing – review & editing, Writing – original draft, Project administration, Investigation, Funding acquisition, Data curation, Conceptualization. **Yongbin Xu:** Investigation, Formal analysis, Conceptualization. **Weihao Chen:** Investigation, Formal analysis. **Jianjun Wu:** Writing – original draft, Supervision, Project administration, Funding acquisition, Conceptualization. **Yu He:** Writing – review & editing, Writing – original draft, Supervision, Project administration, Data curation, Conceptualization.

## Declaration of competing interest

The authors declare that they have no known competing financial interests or personal relationships that could have appeared to influence the work reported in this paper.

## Data Availability

Data will be made available on request.
